# Phenotypic heterogeneity and evolution of melanoma cells associated with targeted therapy resistance

**DOI:** 10.1371/journal.pcbi.1007034

**Published:** 2019-06-05

**Authors:** Yapeng Su, Marcus Bintz, Yezi Yang, Lidia Robert, Alphonsus H. C. Ng, Victoria Liu, Antoni Ribas, James R. Heath, Wei Wei

**Affiliations:** 1 Institute for Systems Biology, Seattle, Washington, United State of America; 2 Division of Chemistry and Chemical Engineering, California Institute of Technology, Pasadena, California, United State of America; 3 Department of Molecular and Medical Pharmacology, University of California – Los Angeles, Los Angeles, California, United State of America; 4 Department of Medicine, University of California – Los Angeles, Los Angeles, California, United State of America; 5 Department of Surgery, Division of Surgical-Oncology, University of California – Los Angeles, Los Angeles, California, United State of America; 6 Jonsson Comprehensive Cancer Center, University of California – Los Angeles, Los Angeles, California, United State of America; Weizmann Institute of Science, ISRAEL

## Abstract

Phenotypic plasticity is associated with non-genetic drug tolerance in several cancers. Such plasticity can arise from chromatin remodeling, transcriptomic reprogramming, and/or protein signaling rewiring, and is characterized as a cell state transition in response to molecular or physical perturbations. This, in turn, can confound interpretations of drug responses and resistance development. Using *BRAF-*mutant melanoma cell lines as the prototype, we report on a joint theoretical and experimental investigation of the cell-state transition dynamics associated with BRAF inhibitor drug tolerance. Thermodynamically motivated surprisal analysis of transcriptome data was used to treat the cell population as an entropy maximizing system under the influence of time-dependent constraints. This permits the extraction of an epigenetic potential landscape for drug-induced phenotypic evolution. Single-cell flow cytometry data of the same system were modeled with a modified Fokker-Planck-type kinetic model. The two approaches yield a consistent picture that accounts for the phenotypic heterogeneity observed over the course of drug tolerance development. The results reveal that, in certain plastic cancers, the population heterogeneity and evolution of cell phenotypes may be understood by accounting for the competing interactions of the epigenetic potential landscape and state-dependent cell proliferation. Accounting for such competition permits accurate, experimentally verifiable predictions that can potentially guide the design of effective treatment strategies.

## Author summary

Cancer cells exhibit varied degrees of phenotypic heterogeneity. These phenotypes, each of them with unique molecular and functional profiles, display dynamic interconversion in response to drug perturbations, and can evolve to form new drug-tolerant phenotypes. Such phenotypic plasticity, in turn, renders tumor cells extremely difficult to treat. To get a quantitative biophysical understanding of the origins of the phenotypic equilibrium and evolution associated with drug tolerance development in highly plastic patient-derived melanoma cells, we employed joint experimental and computational approaches, using either bulk or single cell measurements as input, to interrogate the epigenetic landscape of the phenotypic evolution. We found that the observed phenotypic equilibria were established via competition between state-dependent net proliferation rates and landscape potential. The results reveal how the tumor cells maintain a phenotypic heterogeneity that facilitates appropriate responses to external cues. They implicate that, in certain phenotypically plastic tumor cells, drug targeting the driver oncogenes may not have sustained efficacy unless the phenotypic plasticity of the tumor is co-targeted.

## Introduction

The phenotypic plasticity of many tumors can confound the identification of effective therapeutic strategies [1–4]. For such tumors, even if the cancer cells are isogenic, the cellular composition can be a heterogeneous mix of different cell states (phenotypes) that exhibit the capacity for dynamic interconversion. Each phenotype can have a characteristic gene expression profile, drug susceptibility, proliferation rate, and metastatic potential [5]. When this heterogeneous population is challenged with a physical or molecular perturbation, the cell states can rapidly evolve [6] to form a new population distribution better suited to survive the challenge. This adaption may proceed without genetic changes [7–9]. Removal of the challenge can lead to recovery of the original population distribution [5, 10, 11]. This behavior bears similarities to that of ‘phenotypic equilibria’ [1, 12, 13]. In those systems, if a subset of this population of microstates is physically separated from a stable, heterogeneous population and allowed to expand in culture, the phenotypic heterogeneity of the original culture will recover. This facile adaptability makes plastic tumors challenging to drug-target, and it highlights the importance of quantitative models that can provide predictive and mechanistic insights into the underlying driving force controlling such behaviors.

Similarities between steady states in nonequilibrium biological systems and perturbation/relaxation scenarios in classical thermodynamics equilibria have prompted investigations into applying physicochemical models for describing phenotype dynamics within an epigenetic landscape [13–16]. Qualitative descriptive models have been explored for many years, but quantitative and predictive models have only been recently explored [14–20]. In one class of studies, epigenetic landscape models are explored, wherein stable cell states are described as local minima (attractors) within a metaphoric energy (or potential) cell-state landscape. In such models, the driving forces that influence the cellular composition and population dynamics are the gradients on that surface. As a result, cells tend to gravitate and remain in the local minima of such landscapes. However, in many other cases, this potential landscape does not predict the observed phenotypic heterogeneity [16], implicating other important factors that can influence the population dynamics are at play.

To address this puzzle, we studied highly plastic patient-derived *BRAF*^*V600E*^ mutant melanoma cell lines as models of cancer cell phenotypic plasticity. The high rate of both response [21] and resistance development [10] of *BRAF*-mutant melanoma patients to BRAF inhibitor (BRAFi) treatment has made such cell lines important models for understanding challenges associated with targeted inhibitors [9, 10]. BRAFi can trigger a series of nongenetic cell state changes along the melanocytic lineage towards drug-tolerant and eventually drug resistant states through epigenetic reprogramming. These include the transition of drug-sensitive melanocytic cancer cells into a drug-tolerant neural crest-like phenotype, which, under continued BRAF inhibition, can eventually transition into a fully drug-resistant, invasive mesenchymal-like phenotype [5, 9–11]. The cell biology of this BRAFi-induced phenotypic evolution has been extensively characterized [5, 22], and shown to correlate with what is observed in patient biopsies [9–11, 22]. However, a quantitative biophysical understanding of this type of epigenetic plasticity has not been fully explored.

To this end, we carried out two sets of experiments, integrated with two theoretic approaches, on phenotypically plastic *BRAF*^*V600E*^ mutant melanoma cell lines. At the macroscopic level, we measured a kinetic series of bulk transcriptomes over a 2.5-month course of low dose BRAF inhibition, during which time the cells evolve from a mostly melanocytic, drug-sensitive phenotype to a mesenchymal, drug-tolerant phenotype. This data set provides input into an information-theoretic surprisal analysis [23], which is used to identify the relative free energy-like potential over the entire course of cell state transition from drug response to drug tolerance. We also utilized microscopic inputs from flow cytometry to profile, at the single-cell level, the phenotypic evolution of the same system. These phenotypic evolution dynamic data cannot be described with conventional Fokker-Planck equation but can be well recapitulated using a modified Fokker-Planck-type (FP-type) kinetic model [17, 18, 24] which considered cell-state dependent proliferation differences. The model resolves relative cell state potential and cell-state proliferation differences were quantitatively validated through experiment. We further show that both approaches provide a self-consistent picture in which the combined effects from the relative stability of cellular phenotypes, together with the phenotype-specific net-proliferative rate, act as the drivers to predictably influence the cell population dynamics of drug-induced phenotypic evolution over time. The results provide conceptual guidance for considering effective therapy combinations [5].

## Results

### Surprisal analysis of bulk transcriptome data resolves steady state and time-dependent constraints in the melanocytic to mesenchymal transition

**Fig 1:**
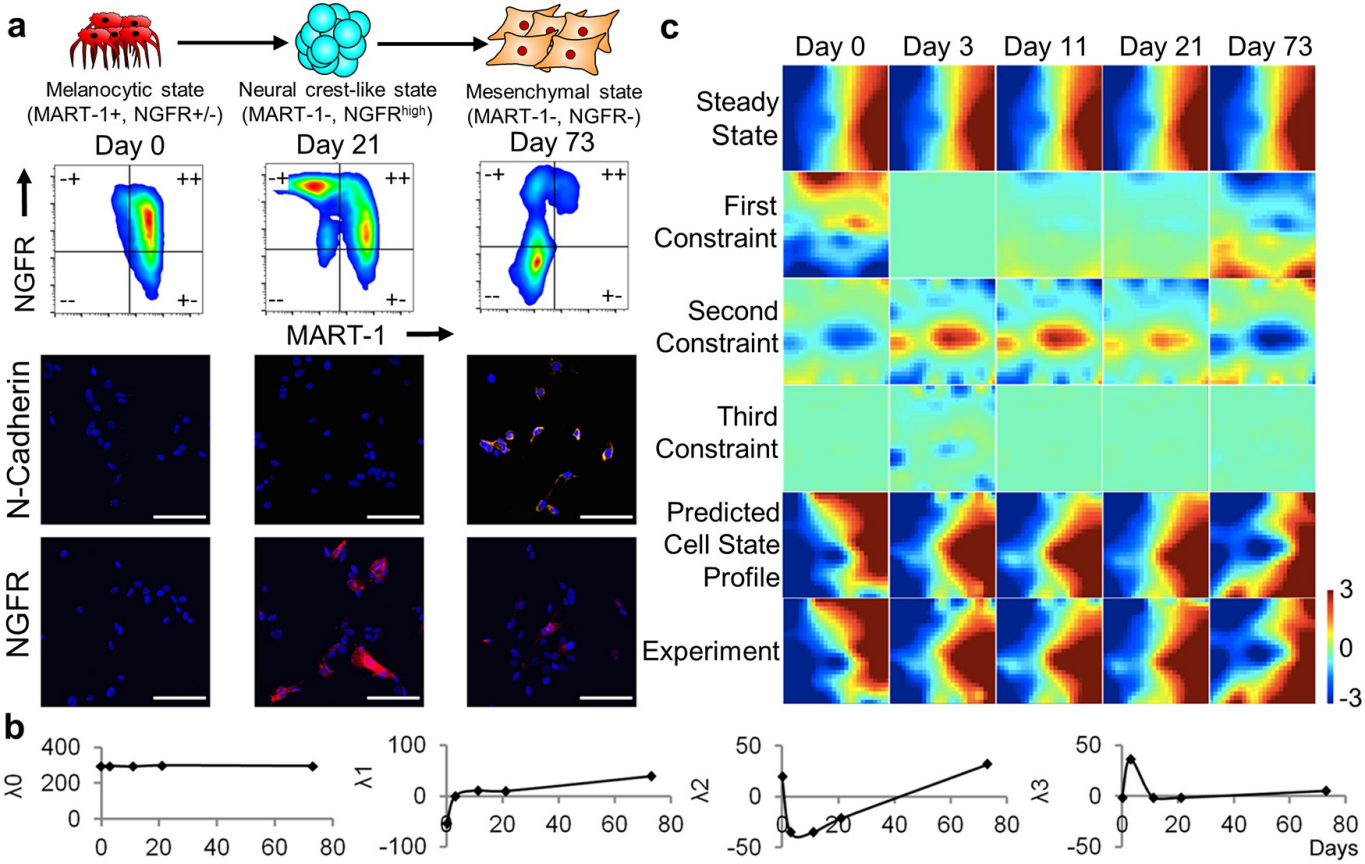
Information theoretic analysis of time-series transcriptome data of a patient-derived BRAF-mutant melanoma cells (M397) treated with a BRAFi. The cells responded to BRAFi by transitioning from a melanocytic to mesenchymal phenotype over the 2.5 month time course. (A) Top: Illustration of the BRAFi-induced phenotype transitions in M397. Middle: Flow cytometry profile of marker proteins MART-1 and NGFR along the course of the transition. Bottom: DAPI, NGFR and N-Cadherin staining of untreated, neural-crest like, and drug-tolerant mesenchymal cells. Scale bar: 100 μm. (B) The amplitude of the steady state and the top three constraints as determined by surprisal analysis of the kinetic series of transcriptome data. (C) The contributions of the steady state and 3 constraints to gene expression are visualized using a self-organizing map to divide the measured transcript levels into 625 (25×25) “miniclusters”. Each minicluster of genes is mapped onto the same pixel in each map. The predicted cell state profile appears as the sum of the steady state and the top three constraints.

We used two patient-derived *BRAF*^*V600E*^ mutant cell lines (M397 and M229) with a prominent melanocytic to mesenchymal phenotypic evolution induced upon BRAF inhibition (Fig 1A and S1 Fig) [5]. We characterized this process by both a bulk transcriptome profiling (S1 Table) and a flow cytometry phenotyping using two protein markers (MART-1 and NGFR) that are established cell phenotype markers for this system [5, 9, 25]. The transcriptome was measured at Day 0 (D0), which served as an untreated control, and at a set of time points following BRAFi (vemurafenib) treatment (S2 Fig). Following drug exposure, the relative location of the binning of cell populations expressing different levels of the two markers followed a counterclockwise transition trajectory around the flow cytometry plots (S1(A) Fig), moving from the melanocytic phenotype (MART-1^pos^) towards a transiently enriched slow-cycling neural crest (MART1^neg^/NGFR^high^) population around day15 to day20 (D15–D20), and eventually terminating at a mesenchymal (MART1^neg^/NGFR^neg^) phenotype at around day62 (D62) with elevated expression of the mesenchymal marker N-cadherin (Fig 1A). This drug-resistant population stably persisted with extended drug treatment beyond D62 (S1(A) Fig). A similar transition trajectory was also observed for M229 (S1(B) Fig). These drug-induced phenotypic transitions agree with previous literature [5, 22].

To assess the overall stability and transcriptomic eigenpatterns associated with the cell population distributions at various time points across the drug-induced phenotypic evolution, we first applied surprisal analysis (Eq 1) to the transcriptome time series. Surprisal analysis extends the principles of maximum entropy and was initially formulated to understand the dynamics of nonequilibrium systems [26]. Using the method of Lagrange multipliers, it seeks the maximum entropy of molecules and identifies the global steady state with minimal free energy, as well as a series of time-dependent constraints that prevent the nonequilibrium system from reaching the global steady state [16, 23, 26, 27]. Surprisal analysis has been extended to characterize biological processes in living cells, where it assesses the maximum entropy of the biomolecules within the cell ensemble through using a simplified approximation of quantum state distributions of the molecular species [23]. Consequently, for a system with kinetic transcriptome data as input, it can extract the time-independent gene expression baseline (the global steady state), as well as a series of gene expression modules (constraints) that evolve with time [16, 23, 26, 28]. A full derivation and thermodynamic interpretation of Eq 1 is provided within the supplementary materials of previous reports [23, 28].

$$\underset{\begin{array}{c} \text{measured}\;\text{expression}\\ \text{level}\;\text{of}\;\text{transcript}\;i\\ \text{at}\;\text{time}\;\text{t} \end{array}}{\underset{︸}{\text{ln}{Χ}_{i}(t)}}=\underset{\begin{array}{c} \text{global}\;\text{steady}\;\text{state}\\ \text{expression}\;\text{level}\;\text{of}\\ \text{transcript}\;i \end{array}}{\underset{︸}{\text{ln}{Χ}_{i}^{0}(t)}}-\underset{\begin{array}{c} \text{deviation}\;\text{terms}\;\text{from}\;\\ \text{the}\;\text{global}\;\text{steady}\;\text{state}\;\\ \text{of}\;\text{transcript}\;i\;\text{(contrainsts)} \end{array}}{\underset{︸}{\sum_{j}\overset{\begin{array}{c} \text{state}\;\text{variable}\;\text{of}\;\\ \text{constraint}\;j\;\text{at}\\ \text{time}\;\text{t} \end{array}}{\overset{︷}{\lambda_{j}(t)}}\overset{\begin{array}{c} \text{contribution}\\ \text{of}\;\text{transcript}\;i\\ \text{to}\;\text{constraint}\;j \end{array}}{\overset{︷}{G_{ij}}}}}$$ (1)

In Eq 1, *X*_*i*_(*t*) is the measured level of transcript *i* at time *t*. This is considered to be the global steady-state level of transcript *i* ($\text{ln}\left(X_{i}^{0}\left(t\right)\right)=-\lambda_{0}G_{i0}$), modified by the sum of the contributions arising from the constrained processes. The global steady state resolved by surprisal analysis is the cellular state with maximum entropy. If there were no constraints acting upon the cells, then Eq 1 predicts that the cells would be in the global steady state. However, there are non-zero constraints (with amplitudes given by the λ_j_ values), which are biological processes that move the system away from the global steady state. Transcripts associated with a constraint are identified through Eq 1 as lowering the entropy of the system, presumably to maintain one or more biological functions. Data mining the set of transcripts associated with a given constraint can provide insight into those biological functions. Although we do not impose the condition that *λ*_*0*_ is time-independent, we neither expect nor find time-dependence (the *λ*_*0*_ variation is <0.7%) (Fig 1B).

To capture the time evolution of the drug-treated cells, each constrained process is represented by a time-dependent amplitude *λ*_*j*_(*t*) and constraint-specific contributions from each transcript *G*_*ij*_. In principle, analysis of the transcriptomic data across the time series from D0 to D73 could resolve several constraints, but we resolve only three for M397 (S3 and S4 Figs). This is illustrated in Fig 1C, where we represent the whole transcriptome data as a self-organized map (SOM) [29]. The map structure is determined using all data sets. Each tile represents a minicluster of genes with similar expression kinetics. Gene clusters with related expression kinetics are placed close together, while clusters exhibiting very different kinetics are placed far apart. The tile color encodes the average expression level of the genes in that minicluster at a given time point. For SOMs representing a specific constraint, that average gene expression level is also weighted by the participation of the genes in the constraint, as determined from Eq 1. The gene expression profile for the global steady state remains unchanged throughout the transition, while the differentially expressed genes (termed eigengenes elsewhere [30]) specific to constraints *λ*_1_, *λ*_2_ and *λ*_3_ vary with time. Summing the global steady state and the three constraints reproduces the map of the measured transcriptome, indicating that, within the noise level of the data, the three major constraints are sufficient to accurately recapitulate gene expression levels globally across the transition. (Fig 1C and S5 Fig).

**Fig 2:**
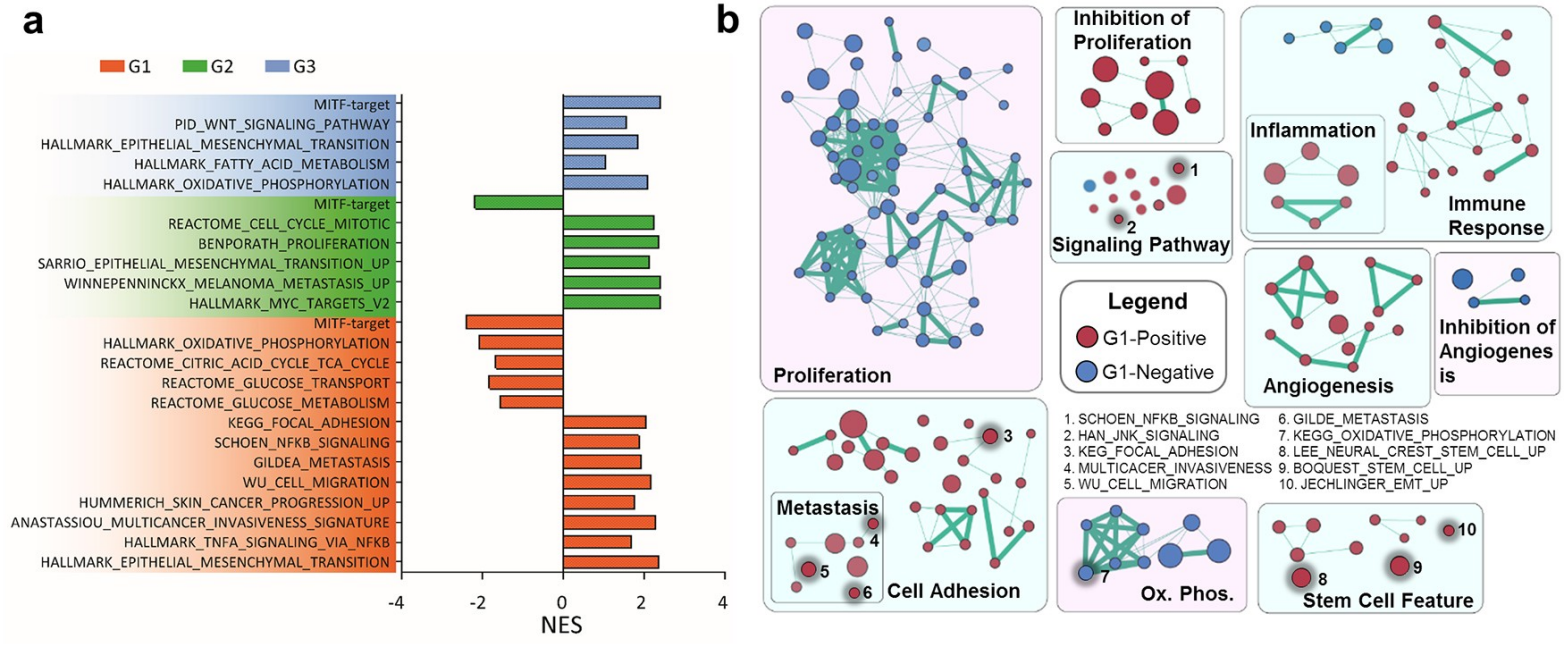
Gene set enrichment of the three constrained processes for the phenotypic and functional changes over the drug-induced phenotypic evolution. (A) List of relevant enriched gene sets, and their relative association with each of the top three constraints. All these gene sets exhibit a nominal p value < 0.05. (B) Cytoscape map that annotates the enriched gene sets associated with the G1 constraint with respect to their functional categories. Enriched gene sets are represented by nodes, which are grouped and annotated based on gene similarity within each gene set. The size of each node is proportional to the total number of genes within each gene set. The edge thickness is proportional to the number of shared genes between gene sets. Red (blue) gene sets are positively (negatively) correlated with G1. Gene sets with similar functions are boxed together with the group name overlaid. Ten specific gene sets are highlighted with thicker outline, and numbered. For example, gene sets 1 and 2 are labeled within the ‘signaling pathways’ box. The corresponding names for those numbered gene sets are provided in the key.

The major biological processes involved in each constraint, at a given time point, can be inferred by enrichment on the gene lists ranked by the constraint-specific contributions from each gene *G*_*ij*_ (Fig 2A, S4 Fig and S2 Table), and by the time-dependent amplitude *λ*_*j*_(*t*) of that constraint. The first constraint shows monotonically increased amplitude (*λ*_1_) along the course of the transition (Fig 1B), with up-regulated mesenchymal signatures, migration, invasiveness and metastasis features, as well as NFκB signaling (*G*_*1*_ positive processes). It also reflects reduced glucose uptake and metabolism, MITF activity, and oxidative phosphorylation (*G*_*1*_ negative processes) (Fig 2A). Constraint 2 contains similar transcriptional signatures, but its amplitude (*λ*_2_) drops after 3 days of BRAFi exposure and slowly increases at later times (Fig 1B). It points to an elevated MITF activity (*G*_*2*_ negative process) and reduced cellular proliferation (*G*_*2*_ positive process) at day-3. This is consistent with previous observations that a brief BRAFi exposure can induce melanocytic differentiation and increased BRAFi sensitivity [31, 32]. The third constraint mainly involves oxidative phosphorylation and the TCA cycle, and has a near zero amplitude except for day 3 (Fig 1B), implying that initial BRAFi exposure leads to a sharply altered metabolic program. The three major constraints associated with M229 displayed similar dynamics and are functionally similar to those in M397 (S4 Fig), confirming the robustness of the BRAFi induced melanocytic to mesenchymal transition. To get a comprehensive view of the enriched transcriptional program, we plotted the enrichment maps of the GSEA results with respect to relevant gene function categories and highlighted representative gene sets in these categories (Fig 2B and S6 Fig). Overall, these transcriptional signatures are wholly consistent with previous reports [5, 9–11, 33], testifying the validity of our cell line model for recapitulating the known biology of the transition and confirming the power of surprisal analysis for dissecting the underlying biology of the transition.

### Fokker-Planck modeling of phenotypic evolution with single-cell flow cytometry phenotyping failed in recapitulating the evolution dynamics

The same biological system was further characterized at the single-cell level using flow cytometry analysis of the established cell-state markers: NGFR and MART-1. The temporal transcriptomic signatures resolved by surprisal analysis result from the dynamics of the BRAFi-induced phenotypic evolution that can be characterized by MART-1 and NGFR marker proteins [5, 9, 25]. As shown in our previous report, these two marker proteins can yield the identical phenotypic classification to that of the whole transcriptome data [5]. Therefore, they can be used as robust phenotype markers during the course of the drug-induced transition (Fig 1A and S1 Fig).

To model the single cell data, we conceptualize cell population distributions as single cells moving on a configuration space delineated by the marker proteins. In this space, cell states correspond to stable or metastable attractors of a hypothetical potential landscape [34]. The dynamics of the protein markers for a single cell can be described by the Langevin type equation *d***z** / *dt* = **μ(z)** + *ζ*, where **z** is the concentration vector of the protein markers (*z*_*1*_, …,*z*_*N*_), **μ(z)** is a drift vector in concentration space that describes all of the deterministic (non-random) dynamics and can be determined by the gradient of the potential landscape. The term *ζ* is the white noise term from random fluctuations in protein expression: 〈*ζ*(*t*)*ζ*(*t*′)〉 = 2**D** *δ*(*t*–*t*′) where **D** is the diffusivity tensor measuring the amplitude of those fluctuations [18].

The potential landscape of a cellular system is context-specific. We hypothesized that drug treatment altered the original drug naïve landscape into a new landscape, which in turn yielded relaxation dynamics as each cell adjusts to this new drift field, potentially with motions towards new attractor states.

**Fig 3:**
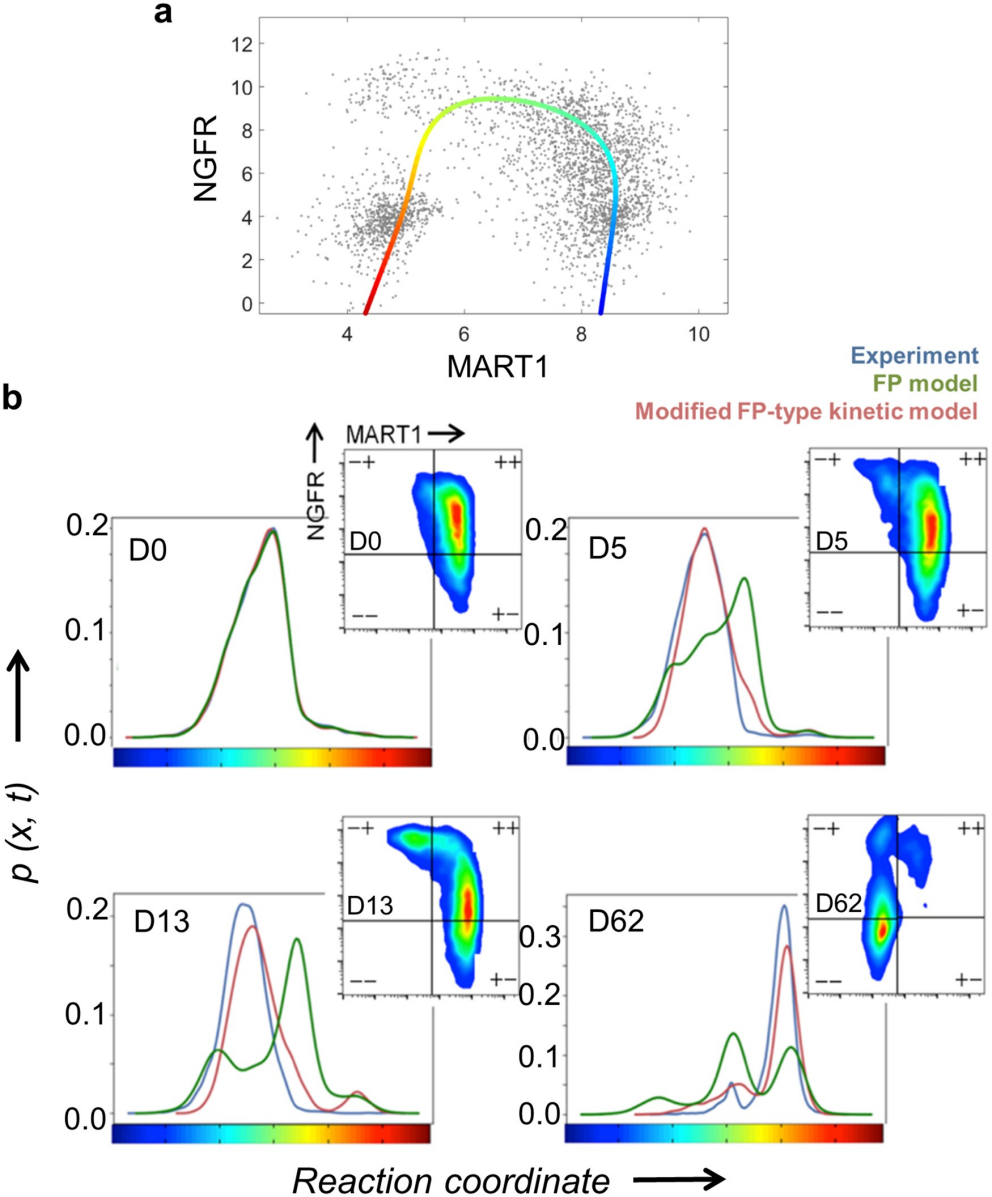
Single-cell flow cytometry analysis of the phenotypic evolution of the M397 cells from melanocyte to mesenchymal under BRAFi treatment, and results of Fokker-Planck-type kinetic model. (A) A reaction coordinate (x), represented as a solid line that evolves from blue (for melanocytic phenotypes) to red (mesenchymal phenotypes), is fitted to the flow cytometry data across all time points. (B) The measured and predicted cell probability density distribution along the reaction coordinate x at representative time points over the transition. Blue line: Experimentally measured distribution of cells. Green line: predicted cell distribution using the original Fokker-Planck model. Red line: predicted distribution from the modified kinetic model that includes a state-dependent cell net growth rate.

Analyzing the dynamics arising from a multi-dimensional drift field is, in general, an intractable problem. However, the flow cytometry trajectory (Fig 1A and S1 Fig) upon BRAF inhibition suggested the simplification that cell populations may be considered to evolve along a linear chain of a limited number of cell states. Therefore, for computational convenience, we projected the protein concentration vectors of the two dimensional (2D) flow cytometry data into a one-dimensional (1D) representation where the cell populations were constrained to move along in this characteristic 1D trajectory (Fig 3A). The distance along the trajectory *x = x(***z***)* serves as an effective reaction coordinate of the phenotypic evolution (see Methods).

The flow cytometry data do not track an individual cell stochastic trajectory, but rather give statistical snapshots of marker protein expression levels across single cells. Thus, it is natural to transform the single-cell Langevin equation into the Fokker-Planck equation for resolving the time-dependent probability distribution *p(x*,*t)* along the reaction coordinate [35]:

$$\frac{\partial p(x,t)}{\partial t}=-\frac{\partial }{\partial x}\left[\mu (x)p(x,t)\right]+\frac{\partial^{2}}{\partial x^{2}}\left[Dp(x,t)\right]$$ (2)

Here, drift term *μ(x)* implies that motion along *x* is influenced by a potential landscape. *D* is a diffusivity that is assumed, for simplicity, to be a constant independent of *x* or drug treatment. Even in cases where the diffusivity depends on the reaction coordinate *x*, a Fokker-Planck (FP) equation with constant diffusivity can be obtained by a simple coordinate transformation as shown in Ref. [17].

Because the dynamics under consideration are 1D, the drift *μ(x)* can always be presented as the derivative of a scalar potential $U(x)=-\int_{0}^{x}\mu (y)dy$. This, in turn, is exactly related to the steady state solution of Eq 2 through a Gibbs relation as limt→∞p(x,t)=p(x)∞=Cexp(−2U(x)/D) where *C* is a normalization constant. Therefore, one can determine (up to proportionality to *D*) the potential *U* from measurement of the steady state distribution *p*_*∞*_*(x)* as U(x)=−(D2)lnp(x)∞. This FP approach has been successfully applied to understanding the population heterogeneity of model biological systems [17, 18]. Here, we used a variation of this method to measure the diffusivity *D* = 0.35*q*^2^ / day (*q* the unit length of the reaction coordinate) from sorting-relaxation experiments in the drug-naïve condition (S7 Fig, See Materials and methods for details). Given this *D* and flow cytometry measurements of the final steady state distribution *p*_∞_(*x*) upon prolonged drug exposure, we inferred the potential *U(x)*, and equivalently the drift *μ(x)* consistent with this model.

To test the validity of the FP model, we performed direct numerical simulation of the FP equation with the inferred *μ(x)*, the diffusivity *D*, and the measured initial distribution *p(x*,*0)* to calculate the cell population distribution *p(x*,*t)* for subsequent days, which, as shown in Fig 3B (FP model), are in poor agreement with the experiments (green lines in Fig 3B and S8 Fig). The disagreement indicated the existence of extra factors influencing phenotypic transitions which were not considered in Eq 2.

### Modified Fokker-Planck-type kinetic model that incorporates cell-state-dependent proliferation recapitulates the phenotypic evolution and predicts cell-state proliferation rates

We hypothesized that the disagreement with experiments arose because the drug would influence not only the cell phenotypic evolution but also the cell autonomous proliferation and survival. In other words, the cells have drug susceptibilities–as reflected by the net effect of cell proliferation and cell killing–that vary along the reaction coordinate. These factors can also influence the phenotypic compositions, but are neglected in Eq 2. Thus, we modified Eq 2 to include a self-sourcing term:

$$\frac{\partial P(x,t)}{\partial t}=-\frac{\partial }{\partial x}\left[\mu (x)P(x,t)\right]+\frac{\partial^{2}}{\partial x^{2}}\left[DP(x,t)\right]+\alpha (x)P(x,t)$$ (3)

Here the net growth rate *α(x)* (the net effect of cell proliferation and cell killing under drug treatment) was introduced to account for cell state-dependent drug susceptibility. As an additional ansatz, we considered *α(x)* as a double step function taking different values for the intermediate neural crest-like phenotype and late-stage mesenchymal phenotype relative to the early stage melanocytic phenotype. It is worthwhile to note that, with Eq 3, we were no longer working with a probability distribution *p(x*,*t)*, but instead a non-normalized population *P(x*,*t)*. Both the differential drift and self-sourcing term act together to induce the cell number changes that are proportional to the population size of a specific cell state. For direct comparison between the model *P(x*,*t)* and experimentally accessible *p(x*,*t)* from flow cytometry data, we simply factored out the norm ($p(x,t)=P(x,t)/N(t)=P(x,t)/\int_{-\infty }^{\infty }dxP(x,t)$).

In this model, due to the addition of the self-sourcing term, the Gibbs relation between the drug-induced steady state *p*_*∞*_*(x)* and the potential *U(x)* used in our analysis of the original FP equation no longer holds. To determine the parameters for this modified model, we therefore resorted to an unbiased numerical search for *U(x)* and *α(x)* that best fit the experimental data. The model prediction was obtained by numerically simulating Eq 3 with the same experimentally measured diffusivity *D* and the initial distribution *p(x*,*0)* as before, together with all possible *U(x)* and *α(x)* values in the unbiased search. We determine goodness of fit using an un-weighted sum-of-square difference between all the predicted and measured cell population distributions *p(x*,*t)*. In both cell lines, we were able to find one set of *U(x)* and *α(x)* for the modified FP-type kinetic model that produced the best prediction of population distributions over time. When compared to original FP model, the modified model predictions are in much better agreement to experiments (red lines in Fig 3B and S8 Fig). The agreement appears to confirm the validity of the self-sourcing term in Eq 3, but the value of that term can be put to an independent experimental test.

**Fig 4:**
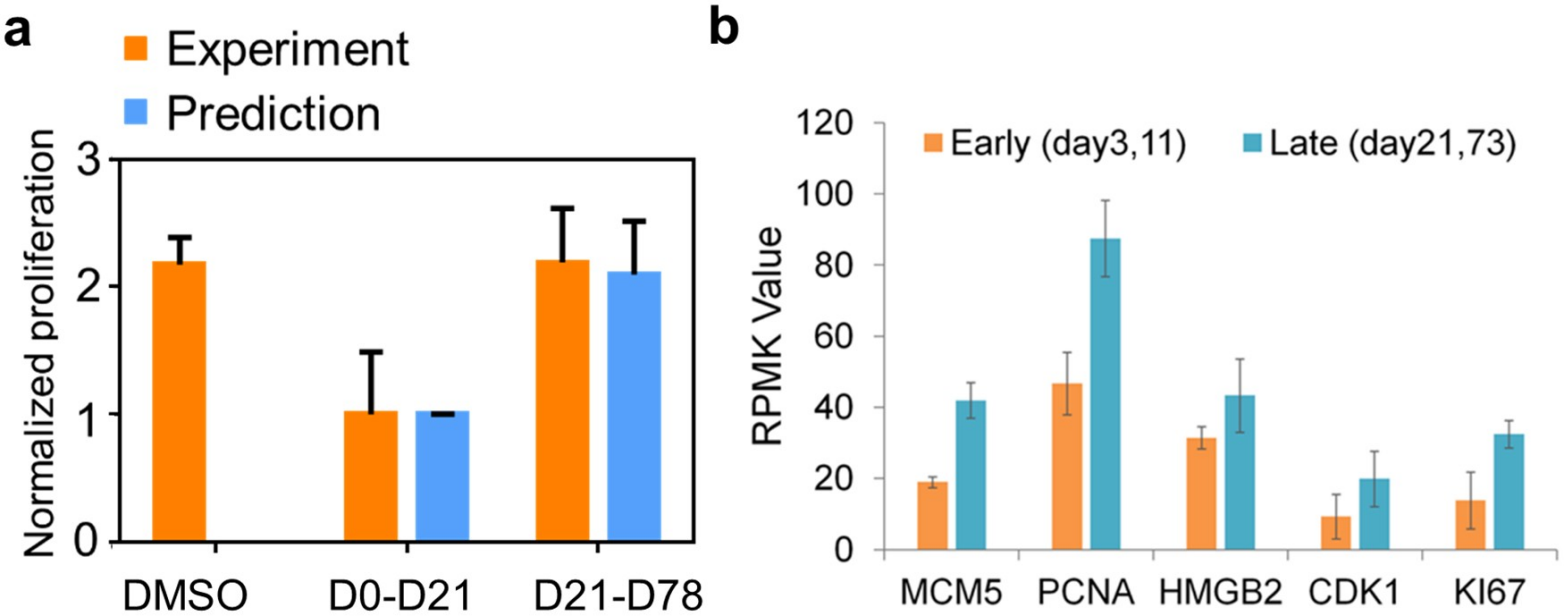
Cell-state-dependent relative net growth rates over the course of the phenotypic evolution for M397 cells. (A) Experimentally measured and predicted cell growth rates (bars). D0-D21 is associated with melanocytic and neural crest-like states, and D21-D78 is primarily associated with the mesenchymal state. Mean values and error bars are defined as mean and s.d., respectively. (B) Expression level of proliferation-related genes after short-term (early) or long-term (late) BRAF inhibition.

We treated the state-dependent net growth rate *α(x)* as a concrete prediction of the model, and found it to be in good agreement with experimentally measured cell growth rates: cell populations containing a higher fraction of the mesenchymal phenotype (day21-78) grow faster than those with a lower fraction (day0-21) (Fig 4, See Materials and methods). The agreements between model-predicted growth rates and experiments (Fig 4) further confirmed the validity of Eq 3 and show that differences in state-dependent growth rates are important in determining the drug-induced phenotypic evolution of the melanoma cells.

**Fig 5:**
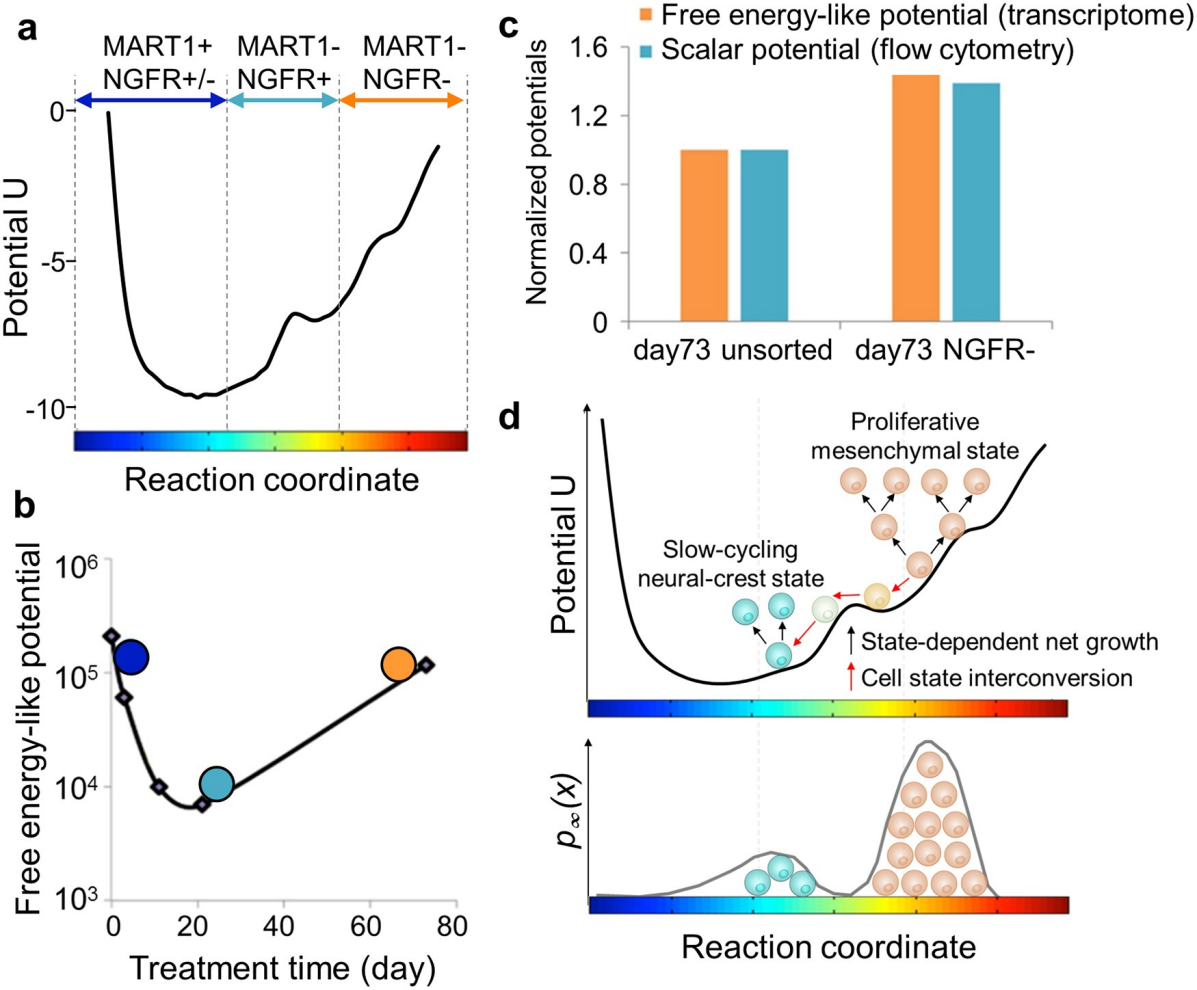
Potential landscapes describing the drug-induced phenotypic evolution from melanocyte to mesenchymal phenotype for M397. (A) The landscape of scalar potential extracted from the modified FP-type (Eq 3) kinetic model. The blue, cyan, and orange arrows indicate regions dominated by melanocytic (MART1+/NGFR±), neural-crest (MART1+/NFGR-) and mesenchymal (MART1-/NGFR-) phenotypes, respectively. (B) The free energy-like potential calculated by surprisal analysis shows the relative cell state stability with respect to the global steady state across different time points. The blue, cyan, and orange circles represent cell populations primarily at melanocytic, neural-crest and mesenchymal phenotypes at the respective time points. (C) Comparison between normalized free energy-like potential (from surprisal analysis, orange bar) and scalar potential (from modified FP-type kinetic model, blue bar) for D73, calculated from transcriptional profiles of unsorted and sorted NGFR-/MART-1- mesenchymal cells. (D) Cartoon illustration of the competition between state-dependent net growth and system stabilization towards the attractor state upon drug treatment.

In addition to predicting proliferation rates, Eq 3 also yielded relative values of the epigenetic potential along the reaction coordinate *U(x)* (Fig 5A and S9(B) and S10(A) Figs), which yields an inference of the stability of different states along the coordinate. The scalar potential landscape was obtained by integration of *μ(x)* from Eq 3 over the reaction coordinate *x*. The shape of the landscape indicates that the intermediate neural crest-like states (NGFR^pos^/MART-1^neg^) are more stable than both the MART-1^pos^ melanocytic state and the mesenchymal-like state (NGFR^neg^/MART-1^neg^), and thus the intermediate states can be considered as an attractor. However, the net growth rate of those intermediate states is relatively low (Figs 4 and 5A), and so the cells do not naturally populate just that state over the course of long-term drug treatment.

### Further confirmation of the concordance of the epigenetic potential landscapes calculated from macroscopic and microscopic inputs

As demonstrated in previous work, surprisal analysis of the bulk RNA-seq data can also define a free energy-like potential corresponding to the drug-induced phenotypic evolution [16, 28]. This potential, for the entire transcriptome of a cell state at time *t*, is relative to the global steady state, and is given by $F(t)=\sum_{j}\lambda_{j}(t)\langle G_{j}\rangle$, where $\langle G_{j}\rangle =\sum_{i}X_{i}G_{ij}$(See Materials and methods for details). It has a direct relationship to the entropy of the transcripts and thus evaluates, at a transcriptional level, the relative stability of a cell state (see Ref. [23] for theoretic details). Here we adopted the same definition to calculate the potential landscape over drug-induced phenotypic evolution in melanoma cells. For M397, this potential landscape calculated from surprisal analysis, similar to the landscape calculated by the modified Fokker-Planck-type (Eq 3) model, indicates that the cells at D11 and D21, with mostly neural-crest like phenotypes are more stable than cells at earlier times (melanocytic phenotypes) or D73 (predominantly mesenchymal phenotype) (Fig 5B). For M229, cells at D21 with mostly the neural-crest like phenotype are also more stable than the cells at D90 (predominantly mesenchymal phenotype) (S10 Fig). Thus, the epigenetic potentials calculated from either surprisal analysis of bulk data or the Fokker-Planck kinetic model from single-cell data yield a consistent picture.

Both analyses indicate that neural-crest like cells are more stable than the mesenchymal phenotype. This prediction was experimentally validated by sorting the mesenchymal (NGFR^neg^/MART1^neg^) subpopulation from the M397 D73 distribution (S11 Fig). We carried out surprisal analysis of transcriptome data from both the segregated mesenchymal subpopulation and the unsorted day-73 population (a mixture of mesenchymal phenotype and neural-crest phenotype). Free energy-like potentials were calculated and found to be consistent with the scalar potentials of both sorted and unsorted populations determined by the modified FP-type kinetic model. The pure mesenchymal phenotype displayed higher potentials than the unsorted cells (Fig 5C). Hence, cell sorting and RNA-seq experiments confirmed the consistence between the two theoretic models, and indicated that the drug-resistant mesenchymal cells are epigenetically unstable relative to the neural crest phenotype.

## Discussion

Heterogeneous cancer cell populations can often exhibit a phenotypic equilibrium and evolution behaviors, meaning that a specific composition comprised of relative abundances of distinct cancer cell phenotypes can be a characteristic of the system, and in the meantime, this characteristic composition will evolve or recover following the application or release of molecular or physical perturbations designed to alter it [2, 5, 12–14]. This can, of course, confound the interpretation of responses to drug treatment, but it also provides a compelling biophysical puzzle. Here we investigated two statistical physics models to help build a predictive picture of such phenotypic equilibria. The models respectively utilize macroscopic and microscopic inputs, and we applied them towards understanding the population dynamics of phenotypically plastic patient-derived *BRAF*-mutant melanoma cancer cells following BRAFi treatment. During a few months period of drug treatment, the cells evolve from drug naïve, drug-sensitive melanocytic-dominated composition to a fully drug-resistant mesenchymal-dominated cell population. In an interesting parallel with state transitions in physical systems, the associated cell state transitions are fully reversible: upon drug removal, the mesenchymal cells revert back to a melanocytic state that is, for all intents and purposes, identical to the initial drug naïve state [5].

The first theoretical model, surprisal analysis, utilizes a bulk transcriptome kinetic series across the drug treatment course to provide a description of the global steady state (the state of maximum entropy) and to identify specific, time-dependent constraints that keep the system from reaching that steady state. The weights of the constrained processes can be utilized to generate a free energy-like potential of the cell-state space sampled during drug treatment [16, 28]. It is worth noting that cells are open systems far from equilibrium. While a significant body of work has demonstrated the apparent parallel between equilibrium and nonequilibrium thermodynamics [36–38], the potential landscape across the cell state evolution in our study is still a metaphor of the real free energy landscape in an equilibrium system. However, the maximum entropy methods can infer the most probable distribution of a probabilistic system regardless of whether or not it is in equilibrium [39]. Surprisal analysis further extends the principles of maximum entropy to understand particularly small systems that are not in thermodynamic equilibrium [23, 26, 40]. Therefore, in analogy to entropy in equilibrium thermodynamics, the entropy (and free energy-like potential) of the cellular transcriptome calculated from surprisal analysis can be used to evaluate the overall stability of a cell state [28, 41].

The second theoretic approach consists of a modified Fokker-Planck-type kinetic model, which takes a kinetic series of single cell flow cytometry data as input. This model considers the Langevin dynamics of self-sourcing single cells moving within a configuration space. That motion is influenced by both (random) diffusion and drift along a potential gradient, thus permitting a potential surface of the traversed cell-state space to also be extracted.

There are two primary considerations that allow results from these two theories to be directly compared. First, the flow cytometry data and the bulk transcriptome data sets capture the same essential biology. This is obviously not always true. However, for this particular case, the cell phenotype markers NGFR and MART-1 used in the single cell assays are known surrogates for drug-induced changes across the whole transcriptome [5]. It also implies that a more selective subset of the transcriptome might equally well recapitulate the underlying biology, which may be assessed by the contribution scores (*G*_*ij*_ values) within each respective constraint. Second, the phenotypic evolution the melanoma cells proceeds stepwise from melanocytes → neural crest → mesenchymal phenotypes. This permits the cell response to BRAF inhibition to be considered as time-dependent motion along a linear reaction coordinate, and provides an equivalence between the Fokker-Planck reaction coordinate and the surprisal analysis time coordinate (Fig 5A and 5B).

We do not directly compare the y-axes of the two landscapes (Fig 5A and 5B), but only the slopes of the curves. The FP scalar potential and the surprisal analysis free-energy like potential have very different origins. The free energy-like potential is derived by comparing transcriptional profiles at each time point with that of the time-independent global steady state. The FP potential is derived from the drift term of Eq 3, and is, in fact, the only term in that equation that needs to be fitted, since both cell proliferation rate and diffusion along the FP reaction coordinate can be experimentally determined. However, both theories predict that the most stable cellular population is a largely neural crest phenotype. Surprisingly, that is not the cell population that is ultimately induced by the long-term drug exposure. That population is dominated by a mesenchymal phenotype with a minor neural crest component, and is arrived at through competing interactions. On the one hand, the neural crest phenotype serves as an attractor, but those cells only slowly proliferate. The higher potential mesenchymal cells are more proliferative and that is the dominating factor. This highlights a major difference between open biological systems and equilibrium thermodynamic systems [42].

The analyses presented here for the *BRAF*-mutant melanoma cells might suggest that identifying drug susceptibilities in each of the cancer cell phenotypes might lead to a more effective therapy. However, such highly plastic cancer cells might eventually switch into cell states that are resistant to even broad combination therapies. A more fruitful approach might be to target those biological mechanisms that underlie the plastic nature of the cells [5, 43].

## Materials and methods

### Ethics statement

Patient-derived melanoma M397 and M229 cell lines were generated from de-identified patient samples with written consent under UCLA IRB approved protocol # 11–003254.

### Patient-derived melanoma tumor models and drug treatment conditions

Cells were cultured at 37°C with 5% CO_2_ in RPMI 1640 with L-glutamine (Mediatech, Inc, Manassas, VA), 10% fetal bovine serum (Omega Scientific Tarzana, CA), and 1% penicillin, streptomycin and fungizone (Omega Scientific Tarzana, CA). Cells were maintained and tested for mycoplasma as previously described [44, 45]. Cell lines were periodically authenticated to their early passages using GenePrint 10 System (Promega, Madison, WI). Presence of mutations in the genes of interest was checked by OncoMap 3 or Iontrone, and was confirmed by PCR and Sanger sequencing as previously described [44, 45].

Vemurafenib (NC0621949, Selleck Chemicals LLC) was dissolved in DMSO at designated concentrations before applying to cell culture media. All cell lines were plated in 10cm dish at 60% confluency and treated with vemurafenib for the specified numbers of days at twice the 50% inhibition concentration (IC50) of each cell line as reported before [5]. At different time points after drug treatment, cells were harvest for RNA-seq and flow cytometry. Cell number was also counted for determining the growth rate. Cell growth rate was fitted as the parameter *α* in the exponential growth curve equation *N*(*t*) = *N*_0_ · 2^(*α*·*t*)^, where *N*_0_ is the cell number at the starting time point, and *N*(*t*) is the cell number at time *t*. Cell numbers counted at day 0, 7 and 21 were used to fit for the proliferation rate at day 0–21 time period, and cell numbers at day 30, 43, 66 and 78 were used to fit for the one at day 21–78 time period.

### Flow cytometry analysis of cell phenotype

At different time points, cells were trypsinized from the dish, spun down and washed with PBS. Cell suspensions were stained for flow cytometry with PE-conjugated NGFR antibody from Biolegend (San Diego, CA). All cells were fixed with Fix-Perm buffer from BD Bioscience (San Jose, CA). Cells were then stained for intracellular Melan-A using FITC conjugated antibody from Santa Cruz (Dallas, TX). Isotypes for mouse IgG1k and mouse IgG1 respectively were used to enable correct gating and to confirm antibody specificity. Blue live-dead staining from Life technologies (Waltham, MA) was used to gate live cell events. 10000 alive events were collected for each sample. Flow cytometry analysis was conducted using LSR-II from BD Biosciences (San Jose, CA), and the data were analyzed using FlowJo software (Tree Star, Inc., San Carlos, California, USA).

### Immunofluorescence imaging

The standard immunofluorescent protocol was implemented using cells grown on the gelatin-coated glass surface. Briefly, 10,000 cells/well were seeded in 96-well glass bottom plates (Greiner Sensoplate Plus, Cat# 655892) coated with 0.1% gelatin solution, and grown in culture media to ~70% confluency. Cells were washed twice in PBS and fixed in 4% paraformaldehyde (PFA) solution for 10 min. After washing twice in wash buffer (0.1% BSA in PBS), cells were blocked and permeabilized in blocking buffer (10% normal donkey serum, 0.3% Triton X-100) for 45 minutes. After removing the blocking buffer, cells were incubated in primary antibody for 4 hours at room temperature. Mouse monoclonal anti-NGFR antibody (BioLegend Cat# 345106 RRID:AB_2152647) or sheep polyclonal anti-N-Cadherin (R&D Systems Cat# AF6426 RRID:AB_10718850) was diluted to 0.25 or 10 μg/mL, respectively, in antibody diluent (1% BSA, 1% normal donkey serum, 0.3% Triton X-100). After washing twice in wash buffer (0.1% BSA in PBS), cells were incubated in secondary antibody for 1 hour at room temperature. Donkey anti-Mouse IgG, Alexa Fluor 647 (Thermo Fisher Scientific Cat# A-31571 RRID:AB_162542) or donkey anti-Sheep IgG Alexa Fluor 594 (Thermo Fisher Scientific Cat# A-11016 RRID:AB_2534083) was diluted to 4 μg/mL in antibody diluent. After washing twice in wash buffer, cells were counter stained for 5 min with 4',6-Diamidino-2-Phenylindole (DAPI) diluted to 1 μg/mL in PBS. After washing twice in PBS, the wells were filled with 78% glycerol.

Fluorescent images were acquired with a Nikon C2plus confocal microscope (Ti) using Plan Apo λ 20× objective (Nikon Inc., Melville, NY). The microscope was controlled by NIS elements AR software (4.51.00) with the following settings: 30 μm pin hole, 12-bit acquisition, 0.62 μm pixel size, 60 gain, and laser power of 5% (405 nm), 0.3% (561 nm), or 0.6% (640 nm). Images were background and contrast adjusted using their respective control wells with no primary antibody staining.

### RNA-seq and transcriptomic data analysis

Cells treated under specified conditions and time periods were trypsinized to harvest for cell pellets. RNA extraction was performed at cell pellets using AllPrep DNA/RNA Mini kit from Qiagen. Bioanalyzer confirmed correct integrity, the library was constructed and Illumina 50 bps single-end RNA-seq data was collected for the samples described. RNA sequencing was performed using 50 bps single end sequencing on the Illumina HiSeq 2500 platform. Libraries were prepared using the IlluminaTruSeq RNA sample preparation kit per the manufacturer’s instructions. Reads were mapped and aligned to the Homo sapiens NCBI build 37.2 reference genome using TopHat2 v2.0.9 [46]. Expression values in fragments per kilobase of exon per million fragments mapped (FPKM) were generated using Cufflinks v2.2.1 program and Cuffnorm to quantify and normalize aligned reads using the geometric library size normalization method [47].

Heatmap and clustering analysis of transcriptomic datasets was performed via MATLAB. Genes are pre-filtered by RPKM value with criteria of average value greater than 0.5 and coefficient of variance greater than 0.15. Filtered gene expression values were standardized across each row (normalized for each individual gene) and represented by a redblue colormap. Hierarchical clustering was performed with average linkage and Euclidean distance metric. Whole transcriptomic dataset and fractions of contributions from each constraints are visualized using self-organized mosaic maps with respect to its control via Gene Expression Dynamics Inspector (GEDI) [29]. Gene Set Enrichment Analysis (GSEA) [48] was performed using GSEA v2.2.3 software with 1000 permutations and weighted enrichment statistics. GSEA enriched gene sets were visualized as interaction networks with Cytoscape [49] and Enrichment Map [50].

### Surprisal analysis and free energy-like potential

Surprisal analysis was applied as described previously [23, 28]. The measured expression level of mRNA *i* at time *t*, *ln X*_*i*_(*t*), was expressed as a sum of a steady state term $ln\;X_{i}^{0}(t)$ and several constraints *λ*_*j*_(*t*)*G*_*ij*_ representing deviations from the steady state. Each deviation term was a product of a time-dependent weight of the constraint *λ*_*j*_(*t*), and the time-independent contribution of the transcript to that constraint *G*_*ij*_.

To implement surprisal analysis, we computed the singular value decomposition (SVD) of the matrix *ln X*_*i*_(*t*). As well described previously [23], the SVD factored this matrix in a way that determined the two sets of parameters that are needed in surprisal analysis: the Lagrange multipliers *(λ*_*j*_*)* for all constraints at a given time point, and for all times and the *G*_*ij*_ (time-independent) transcription patterns for all transcripts *i* at each constraint *j*.

The free energy-like potential calculation based on the surprisal analysis result was implemented as in Ref. [33]. Briefly, The steady-state expression level of transcript *i* at time *t* can be linked to its actual expression level by as $X_{i}^{0}(t)=X_{i}(t)\text{exp}(-\sum_{j}\lambda_{j}(t)G_{ij})$. Therefore, as shown in Ref. [33], surprisal analysis defines the free energy-like potential of a transcript *i* relative to the global steady state at time *t* as $f_{i}(t)=\sum_{j}\lambda_{j}(t)G_{ij}$. Taking all the transcripts into account, the free energy-like potential of the entire transcriptome of a cell state at time *t* relative to the global steady state is given by $F(t)=\sum_{i}X_{i}f_{i}(t)=\sum_{j}\lambda_{j}(t)\langle G_{j}\rangle$, where $\langle G_{j}\rangle =\sum_{i}X_{i}G_{ij}$[16].

Natural log transformed transcriptomic dataset and fractions of contributions from each constraints (*λ*_*j*_(*t*)*G*_*ij*_) calculated from surprisal analysis are visualized using self-organized maps (SOM). Self-organized map visualization of high-dimensional dataset in a form appropriate for human pattern recognition without discarding the global, higher-order information. Here, they present individual samples as a single 2-dimensional heatmap and, at the same time, display high-resolution patterns. Thousands of input genes are assigned to 625 rectangular “tiles” (SOM nodes), each of which represents a mini-cluster of genes, arranged so as to form a pattern within a 2-dimensional mosaic map on the SOM grid. Tile represent most similar clusters will be placed adjacent to each other in the mosaic. Gene Expression Dynamics Inspector (GEDI) package is utilized to implement the SOM visualization [29].

### Modified Fokker-Planck-type kinetic model

The dynamics of the protein markers for a single cell can be described by the Langevin type equation *d***z** / *dt* = **μ(z)** + *ζ*, where **z** is the concentration vector of the protein markers (*z*_*1*_, …,*z*_*N*_), **μ(z)** is a drift vector in concentration space that describes all of the deterministic (non-random) dynamics and can be determined by the gradient of the potential landscape. The term *ζ* is the white noise term from random fluctuations in protein expression: 〈*ζ*(*t*)*ζ*(*t*′)〉 = 2**D** *δ*(*t*–*t*′) where **D** is the diffusivity tensor measuring the amplitude of those fluctuations [18].

In the case of melanocytic to mesenchymal transition, for computational convenience, we projected the protein concentration vectors of the flow cytometry data into a one-dimensional (1D) representation where the cell populations were constrained to move along in this characteristic trajectory. This converted each snap-shot of cell population distribution from the 2D flow cytometry plot onto a one-dimensional distribution along the linear trajectory. More specifically, we reduced the dimensionality of the flow cytometry data by calculating the principle curve of the full set of measurements using the R package princurve. The data points were projected onto the curve, and the distances of these projected points along the curve were used as the one-dimensional data for the two Fokker-Planck models. These data points were converted into probability density functions (PDF) using kernel density estimation.

Consider the fact that flow cytometry data do not track an individual cell stochastic trajectory but rather give statistical snapshots of marker expression levels across many single cells. Thus, it is natural to transform the single-cell Langevin equation into the Fokker-Planck equation for resolving the probability distribution of the protein markers. The 1D coordinate (Fig 3A) is defined as a reaction coordinate *x(z)* such that the Fokker-Planck (FP) equation for the probability distribution *p(x*,*t)* has the following form:

$$\frac{\partial p(x,t)}{\partial t}=-\frac{\partial }{\partial x}\left[\mu (x)p(x,t)\right]+\frac{\partial^{2}}{\partial x^{2}}\left[Dp(x,t)\right]$$ (M1)

Here, *x* is the 1D flow cytometry (FC) coordinate, *D* is a diffusion constant of the cells along *x*, and drift term *μ(x)* implies that motion along *x* is influenced by a potential landscape. In this model, the equilibrium distribution *p*_*∞*_*(x)* = lim_*t→∞*_*p(x*,*t)* and the potential *U*(*x*) = −∫*μ*(*x*)*dx* are connected through the Gibbs relation

$$U(x)=-\left(\frac{D}{2}\right)\text{ln}p_{\infty }(x)$$ (M2)

For the unmodified Fokker-Planck equation, this Gibbs relation was applied to the long-term drug treated cell population distribution data (day78 for M397 and day60 for M229) to infer a potential, whose gradient acted as the drift term driving the dynamic changes of the population distribution. This inferred potential and respective drift term, when coupled with diffusion constant *D* and the initial (day0) population distribution, generated the prediction results in Fig 3B.

With regards to calculating diffusion constant *D* from cell sorting and relaxation experiments, the diffusion coefficient *D* was assumed to be a constant value independent of trajectory position *x* and drug treatment condition for simplicity. Based on this assumption, when calculating the diffusion constant, we used time-series flow cytometry data of cell sorting and relaxation experiments. In these experiments, we sorted out the untreated cells into NGFR^pos^ and NGFR^neg^ subpopulations. Both sorted subpopulations were cultured without drug treatment. At different days after sorting and culturing, the cells were harvested to quantify its abundance of NGFR and MART-1 using flow cytometry as shown S6 Fig.

Consider the fact that variations of proliferation rates are small in the untreated cells, the Fokker-Planck model (Eq M1) was considered valid and this data was used as input to fit the diffusion constant *D*. Varying *D* as a free parameter, the (drug-naïve) potential and hence drift were calculated with Eq M2, using the original, untreated distribution as *p*_*∞*_*(x)*. The Fokker-Planck equation with these parameters was simulated, with the initial condition *p(x*,*0)* set by the sorted population distribution. The simulated data were compared to the measured time-series distributions with an unweighted sum-of-squares measure. This measure was then minimized as a function of *D*, and yielded the best-fit diffusion constant *D* to be 0.35 *q*^*2*^/day where *q* represents the unit length on the flow cytometry coordinate.

For the modified Fokker-Planck-type kinetic model described in Eq (3) where the same reaction coordinate (*x*) as the unmodified equation is applied, the state-dependent proliferation rate *α(x)* was modeled as piecewise-constant with different values for the melanocytic, neural crest, and mesenchymal cell types. The cutoff locations in terms of the reaction coordinate *x* were chosen as the two local minima in an observed PDF with the coexistence of all three subpopulations. One can show that the time evolution of the PDF does not depend on an overall constant shift *α(x)+c*, so we set the proliferation rate of the starting melanocytic state to 0 for convenience, as the melanocytic cells were observed to be cytostatic without significant proliferation or cell death upon drug treatment. This then left the proliferation rates of the neural crest (*α*_*1*_) and mesenchymal cells (*α*_*2*_) as two free parameters.

Because the Gibbs relationship between the long-time density *p*_*∞*_*(x)* and the potential *U(x)* no longer held with this nontrivial proliferation rate, we resorted to fitting a cubic spline interpolation for the drift *μ*(*x*) = −*∂U*(*x*) / *∂x*. Twenty spline points were used, with *x* values uniformly spaced along the curve and *μ* values as free parameters.

Starting with an estimate of *α*_*1*_*(x) = α*_*2*_*(x) = 0* and *μ ~ x*, we calculated the prediction of this model using FiPy to numerically simulate the forward evolution with initial condition *p(x*,*0)* set by the experimentally measured distribution on day-0. To compare with the experimental data, we used the L^2^ norm on the difference between the predicted and experimental probability densities $L={\sum_{i}\int_{-\infty }^{\infty }\left(p_{pred}(x,t_{i})-p_{exp}(x,t_{i})\right)}^{2}dx$ as the goodness-of-fit metric. Gradient descent was performed on the proliferation and drift parameters to determine the best-fit values that minimize *L*. The calculated potential landscape results are robust to small variations in parameters for calculating the principal curve. (S12 Fig).

## Supporting information

S1 Fig Kinetic flow cytometry data of drug-induced phenotypic evolution. A. Cartoon illustration of the BRAFi-induced transition where the melanoma cells take an approximately counterclockwise trajectory around the flow cytometry plot. B. Flow cytometry plots of NGFR and MART-1 protein markers for M397 at a set of the points over the drug-induced phenotypic evolution. Data are represented as a 2-dimensional density plot for each day. As the de-differentiation transition occurs from day 0 to day 78, the cell population moves along a counterclockwise trajectory. C. Flow cytometry plots of NGFR and MART-1 protein markers for M229 at a set of the points over the drug-induced phenotypic evolution.
(PDF)

S2 Fig Heatmap and hierarchical clustering for transcriptomic data of M397 and M229 cells under different drug treatment and/or sorting conditions. A is for M397 and B is for M229. Each Row of the heatmap indicates each gene. Each column is a sample condition, as indicated. Color represents gene expression level, with up-regulated genes colored in red and down-regulated genes colored in blue. Different molecular baselines of the two melanoma cell lines dictate distinct clustering patterns that require Surprisal analysis to resolve the altered molecular features shared by the two cell lines across the transition.
(PDF)

S3 Fig Heatmap visualization of amplitudes for steady state and different constraints across different samples of M397 and M229. M397 data is shown in panel A and that of M229 is shown in panel B. Each row indicates a constraint, with λ_0_ the global stable state. Each column is a sample condition, as indicated. Positive valued constraints are red, and negative are blue.
(PDF)

S4 Fig Comparison of surprisal analysis result between M397 and M229. A. The amplitude of steady state and top three constraints across different time points determined by surprisal analysis of M397 cell line. B. The amplitude of steady state and top three constraints across different time points determined by surprisal analysis of M229 cell line. C. Gene set enrichment of the three constrained processes for the phenotypic and functional changes of M397 (left) and M229 (right) over the drug-induced phenotypic evolution. Each bar represents one enriched gene sets associated with the top three constraints as indicated by their respective colors. Value represents the normalized enrichment score (NES) calculated from GSEA.
(PDF)

S5 Fig Scatter plot comparison of the measured versus the predicted gene expression levels for M397 from surprisal analysis across different time points, using the global stable state and top three constraints. (PDF)

S6 Fig Enrichment map of the enriched gene sets in the second constraint, as identified by GSEA. (PDF)

S7 Fig Cell sorting and relaxation experiments of M397. A. Illustration of cell sorting experiments. Cells cultured without drug treatment are harvested and stained with NGFR antibody. A flow cytometer separates the NGFR+ live cell subpopulations and the sorted cells are then cultured in the same condition as before sorting. The NGFR and MART-1 (not changing) expression levels are measured for subsequent days as the population re-equilibrates towards the unsorted steady state distribution. B. Flow cytometry data of log NGFR level from cell sorting experiment. The relaxation dynamics of the sorted subpopulation is measured using flow cytometry. Dataset illustrated here was later modeled by a Fokker-Planck equation to determine the diffusion constant of the system.
(PDF)

S8 Fig The measured and predicted cell probability density distribution of M229 along reaction coordinate *x* at various time points. Blue line: experimental data distribution. Green line: predicted distribution using the original Fokker-Planck model (FP model). Red line: predicted distribution from the modified FP-type kinetic model that includes a state-dependent net growth rate.
(PDF)

S9 Fig Comparison of potential calculated from unmodified and modified Fokker-Planck-type kinetic models. Potential landscape calculated from unmodified Fokker-Planck model is shown in panel A and the one from modified FP-type kinetic model is shown in panel B.
(PDF)

S10 Fig The potential landscapes describing the drug-induced phenotypic evolution from melanocytic to mesenchymal phenotype for M229. A. Potential landscape extracted from modified FP-type kinetic model. B. The free energy-like potential calculated by surprisal analysis shows the relative change in stability with respect to the global stable state across different time points.
(PDF)

S11 Fig Illustration of cell sorting for NGFR negative phenotype of M397 at day 73. To validate the free energy calculation from the surprisal analysis, pure NGFR-/MART- subpopulation was sorted using flow cytometry for RNA sequencing and compared against RNA-seq from unsorted cells.
(PDF)

S12 Fig Sensitivity analysis of “Principal Curve”. A. Three principal curves calculated with different iteration number. B. Potential U calculated for all three different principal curves.
(PDF)

S1 Table Kinetic RNA-seq data for M397 and M229 cells. (XLSX)

S2 Table The top 100 genes that contribute positively and negatively to the top3 constraints. (XLSX)
