# Supplementary figures and images for: Phenotypic heterogeneity and evolution of melanoma cells associated with targeted therapy resistance
Source: PLoS Comput Biol. 2019 Jun 5;15(6):e1007034. doi: 10.1371/journal.pcbi.1007034 (PMC6576794; doi:10.1371/journal.pcbi.1007034)

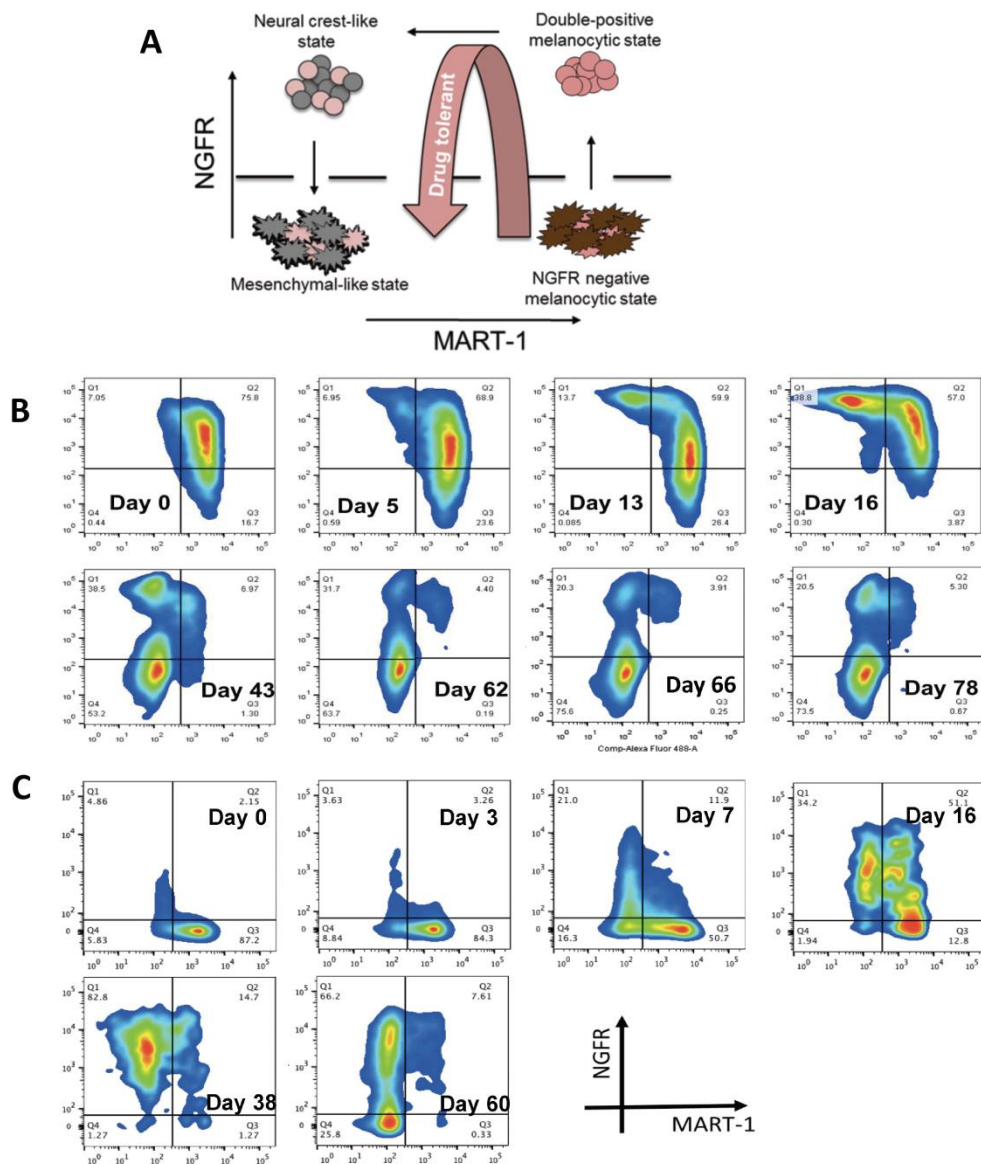

Supplement: S1 Fig — A. Cartoon illustration of the BRAFi-induced transition where the melanoma cells take an approximately counterclockwise trajectory around the flow cytometry plot. B. Flow cytometry plots of NGFR and MART-1 protein markers for M397 at a set of the points over the drug-induced phenotypic evolution. Data are represented as a 2-dimensional density plot for each day. As the de-differentiation transition occurs from day 0 to day 78, the cell population moves along a counterclockwise trajectory. C. Flow cytometry plots of NGFR and MART-1 protein markers for M229 at a set of the points over the drug-induced phenotypic evolution. (PDF) [file pcbi.1007034.s001.pdf]

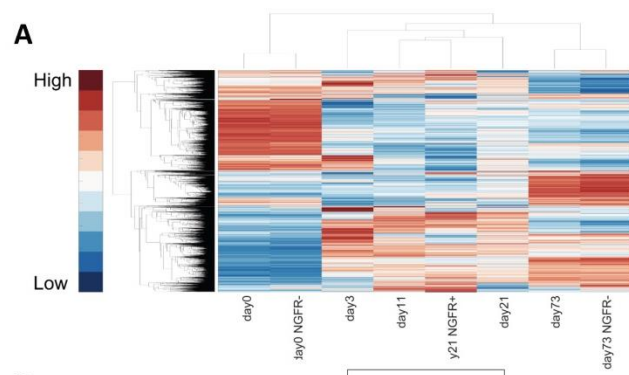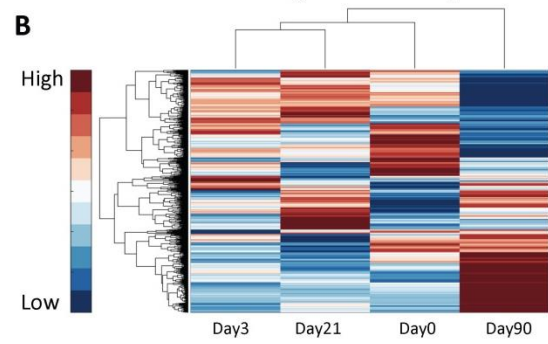

Supplement: S2 Fig — A is for M397 and B is for M229. Each Row of the heatmap indicates each gene. Each column is a sample condition, as indicated. Color represents gene expression level, with up-regulated genes colored in red and down-regulated genes colored in blue. Different molecular baselines of the two melanoma cell lines dictate distinct clustering patterns that require Surprisal analysis to resolve the altered molecular features shared by the two cell lines across the transition. (PDF) [file pcbi.1007034.s002.pdf]

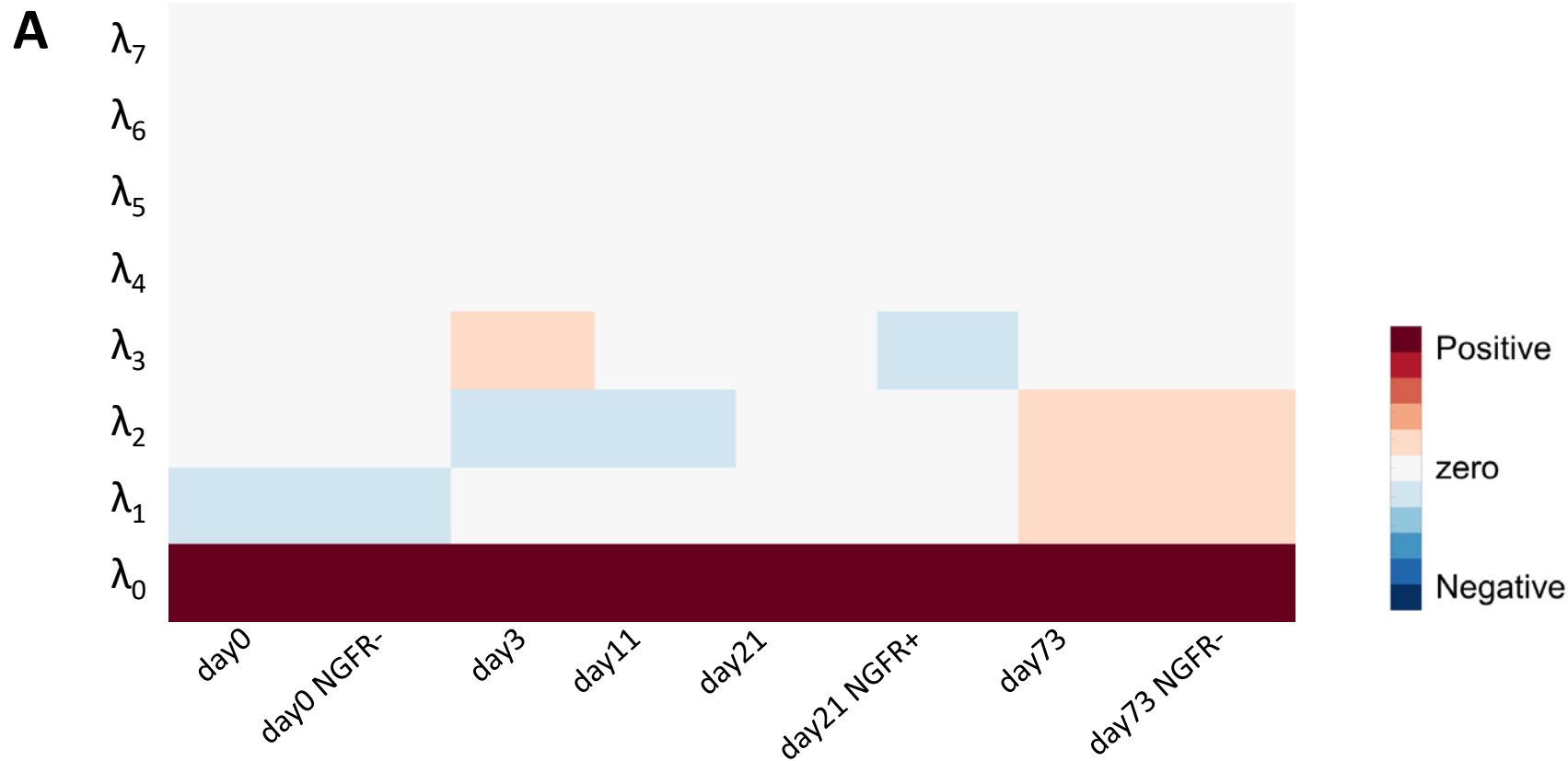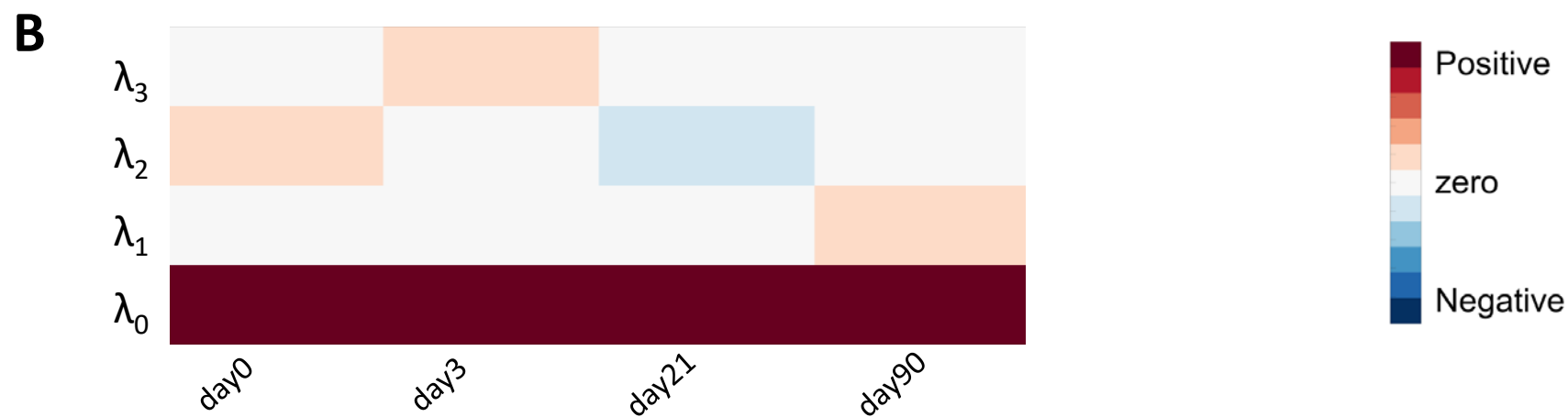

Supplement: S3 Fig — M397 data is shown in panel A and that of M229 is shown in panel B. Each row indicates a constraint, with λ0 the global stable state. Each column is a sample condition, as indicated. Positive valued constraints are red, and negative are blue. (PDF) [file pcbi.1007034.s003.pdf]

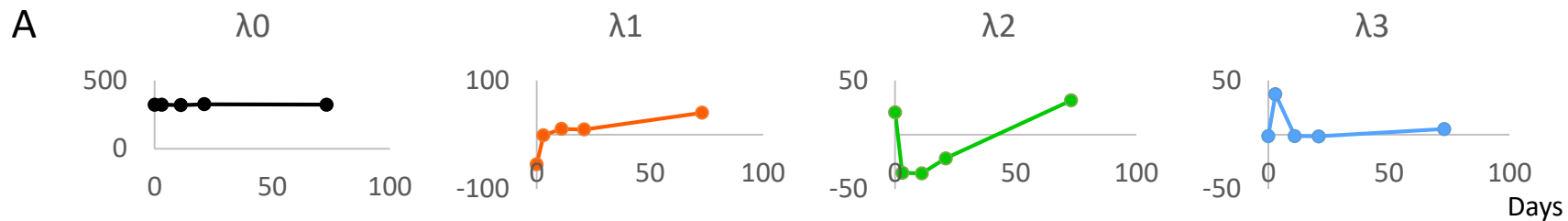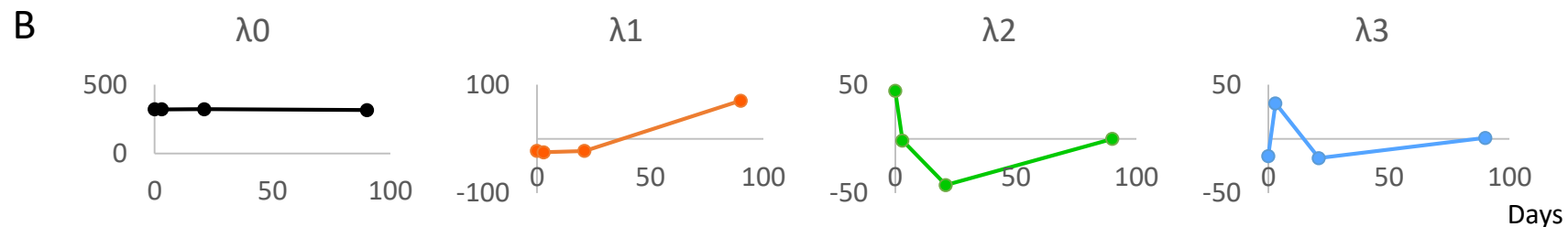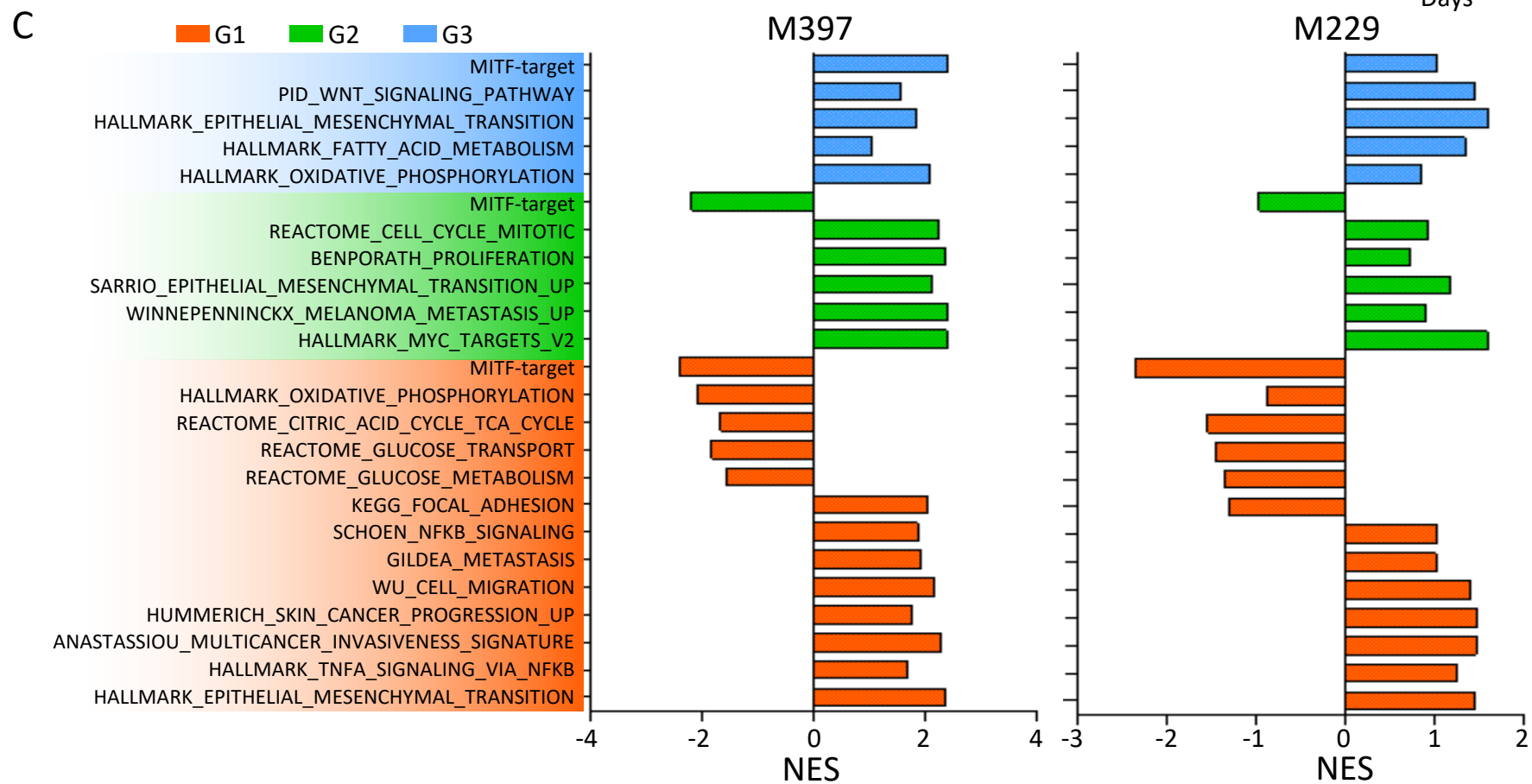

Supplement: S4 Fig — A. The amplitude of steady state and top three constraints across different time points determined by surprisal analysis of M397 cell line. B. The amplitude of steady state and top three constraints across different time points determined by surprisal analysis of M229 cell line. C. Gene set enrichment of the three constrained processes for the phenotypic and functional changes of M397 (left) and M229 (right) over the drug-induced phenotypic evolution. Each bar represents one enriched gene sets associated with the top three constraints as indicated by their respective colors. Value represents the normalized enrichment score (NES) calculated from GSEA. (PDF) [file pcbi.1007034.s004.pdf]

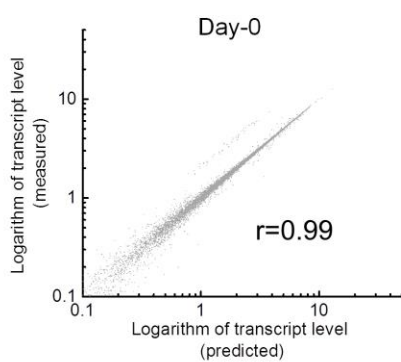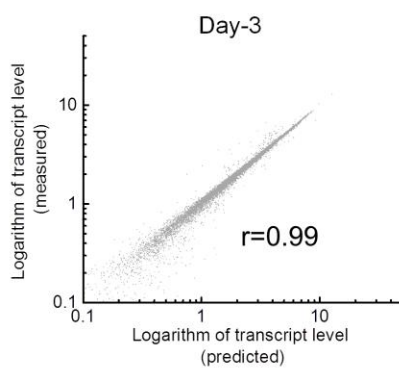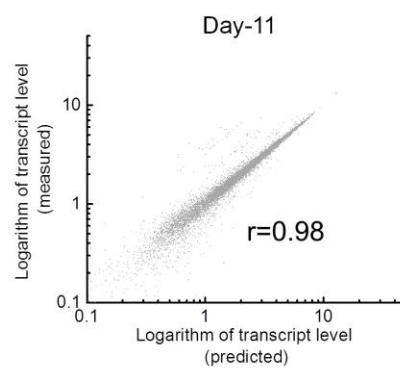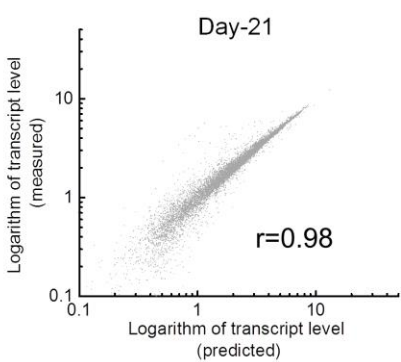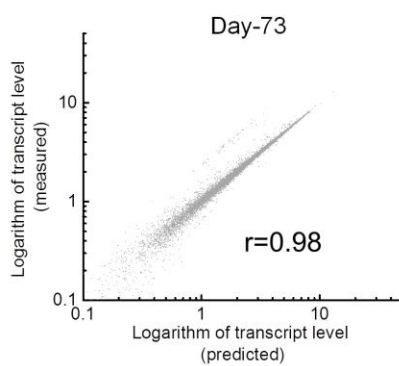

Supplement: S5 Fig — (PDF) [file pcbi.1007034.s005.pdf]

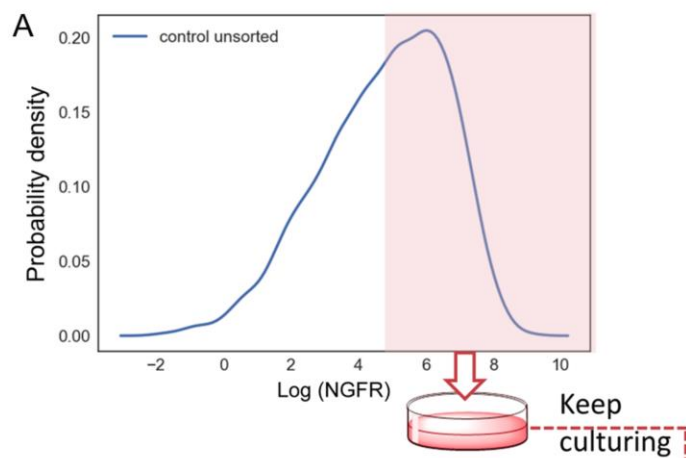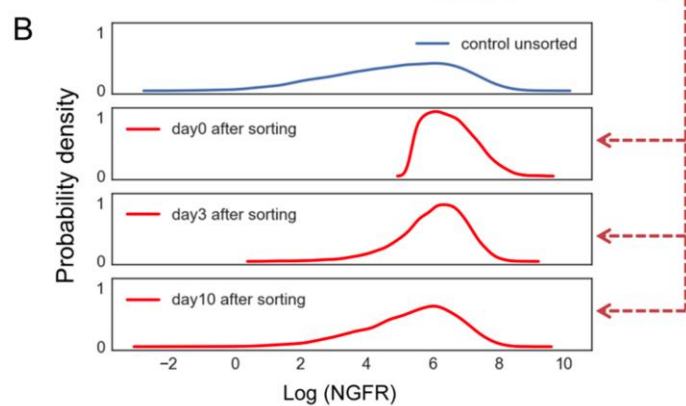

Supplement: S7 Fig — A. Illustration of cell sorting experiments. Cells cultured without drug treatment are harvested and stained with NGFR antibody. A flow cytometer separates the NGFR+ live cell subpopulations and the sorted cells are then cultured in the same condition as before sorting. The NGFR and MART-1 (not changing) expression levels are measured for subsequent days as the population re-equilibrates towards the unsorted steady state distribution. B. Flow cytometry data of log NGFR level from cell sorting experiment. The relaxation dynamics of the sorted subpopulation is measured using flow cytometry. Dataset illustrated here was later modeled by a Fokker-Planck equation to determine the diffusion constant of the system. (PDF) [file pcbi.1007034.s007.pdf]

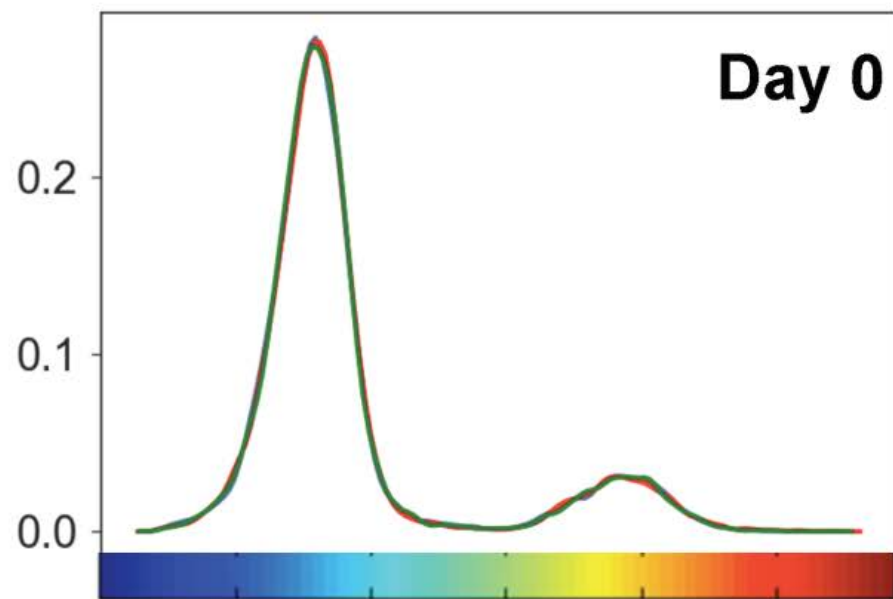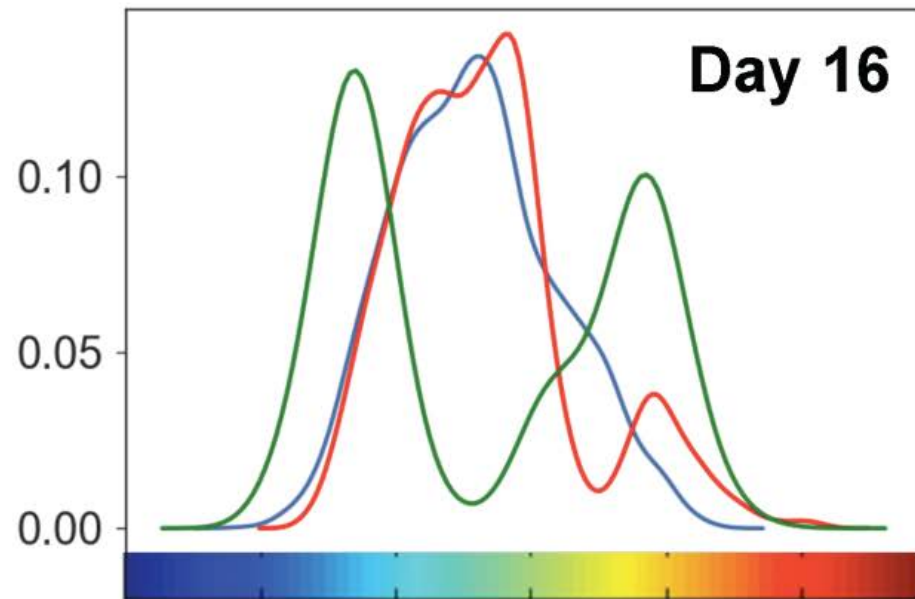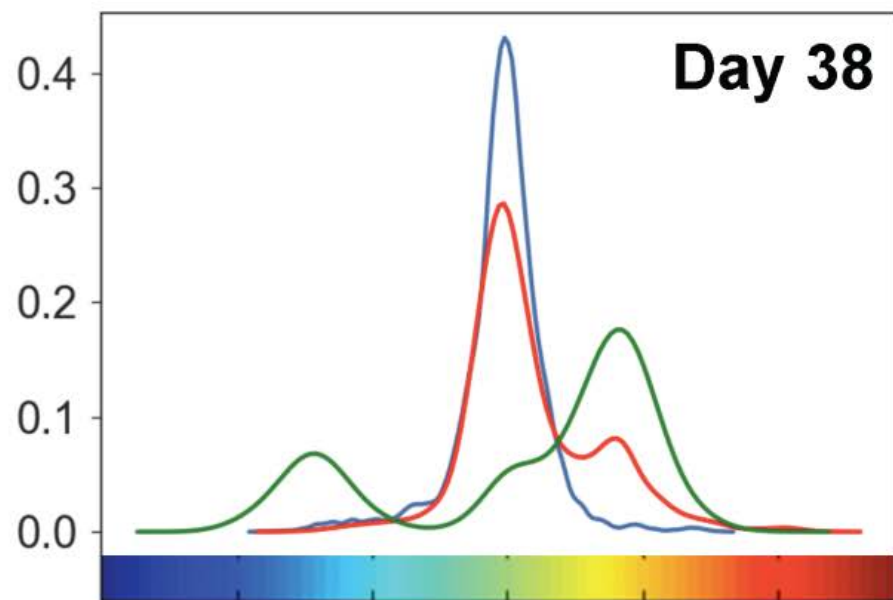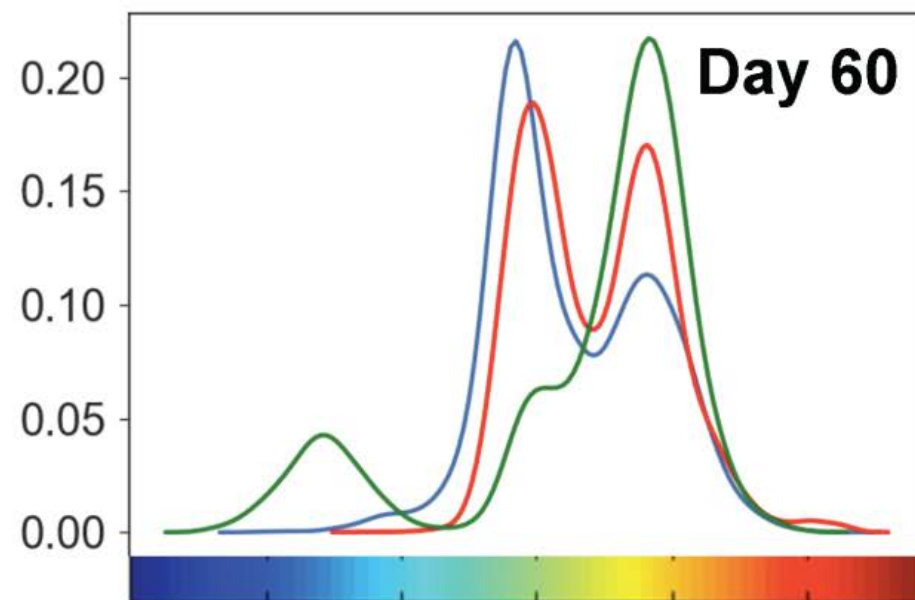

— Experiment

— Unmodified FP model

— Modified FP-type kinetic model

Supplement: S8 Fig — Blue line: experimental data distribution. Green line: predicted distribution using the original Fokker-Planck model (FP model). Red line: predicted distribution from the modified FP-type kinetic model that includes a state-dependent net growth rate. (PDF) [file pcbi.1007034.s008.pdf]

**A****Unmodified FP model**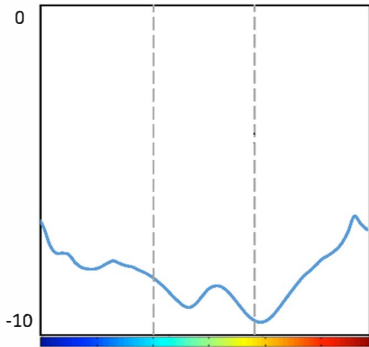**B****Modified FP-type kinetic model**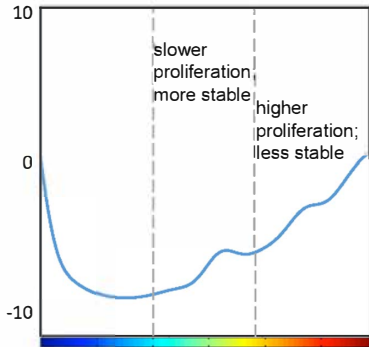

Supplement: S9 Fig — Potential landscape calculated from unmodified Fokker-Planck model is shown in panel A and the one from modified FP-type kinetic model is shown in panel B. (PDF) [file pcbi.1007034.s009.pdf]

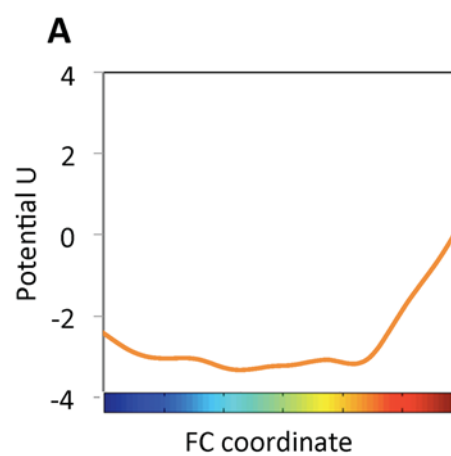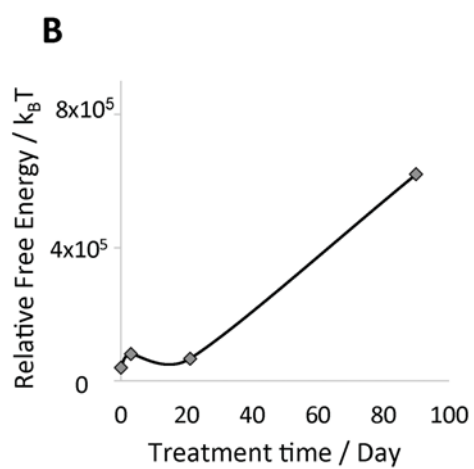

Supplement: S10 Fig — A. Potential landscape extracted from modified FP-type kinetic model. B. The free energy-like potential calculated by surprisal analysis shows the relative change in stability with respect to the global stable state across different time points. (PDF) [file pcbi.1007034.s010.pdf]

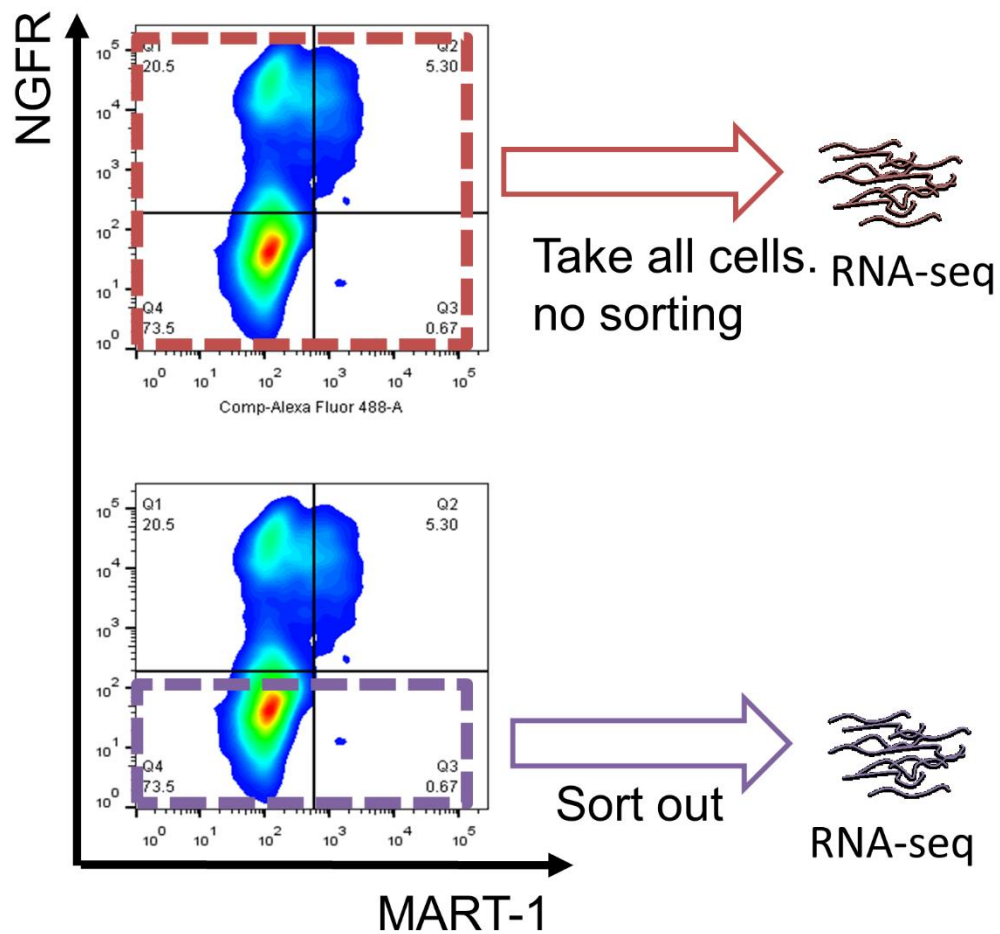

Supplement: S11 Fig — To validate the free energy calculation from the surprisal analysis, pure NGFR-/MART- subpopulation was sorted using flow cytometry for RNA sequencing and compared against RNA-seq from unsorted cells. (PDF) [file pcbi.1007034.s011.pdf]

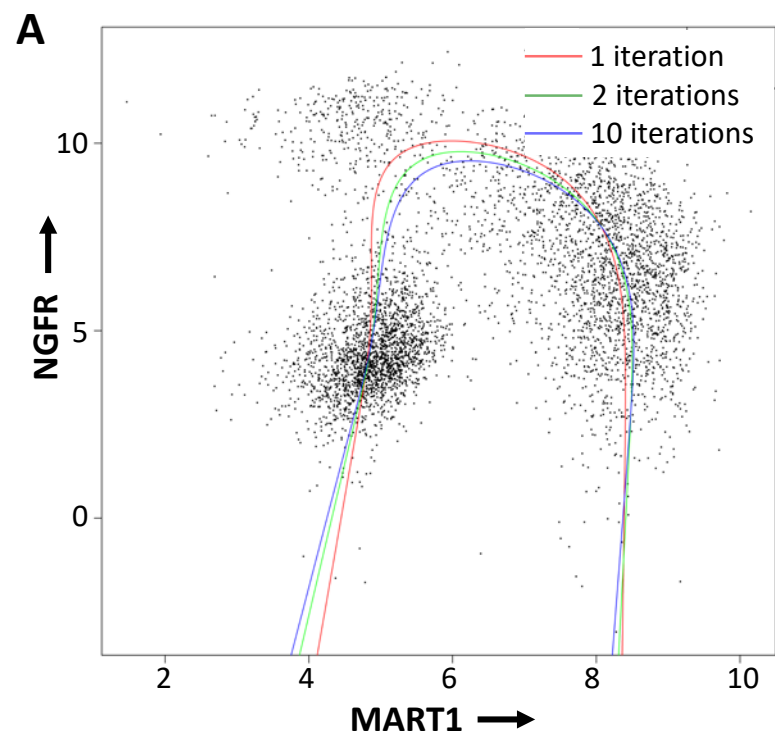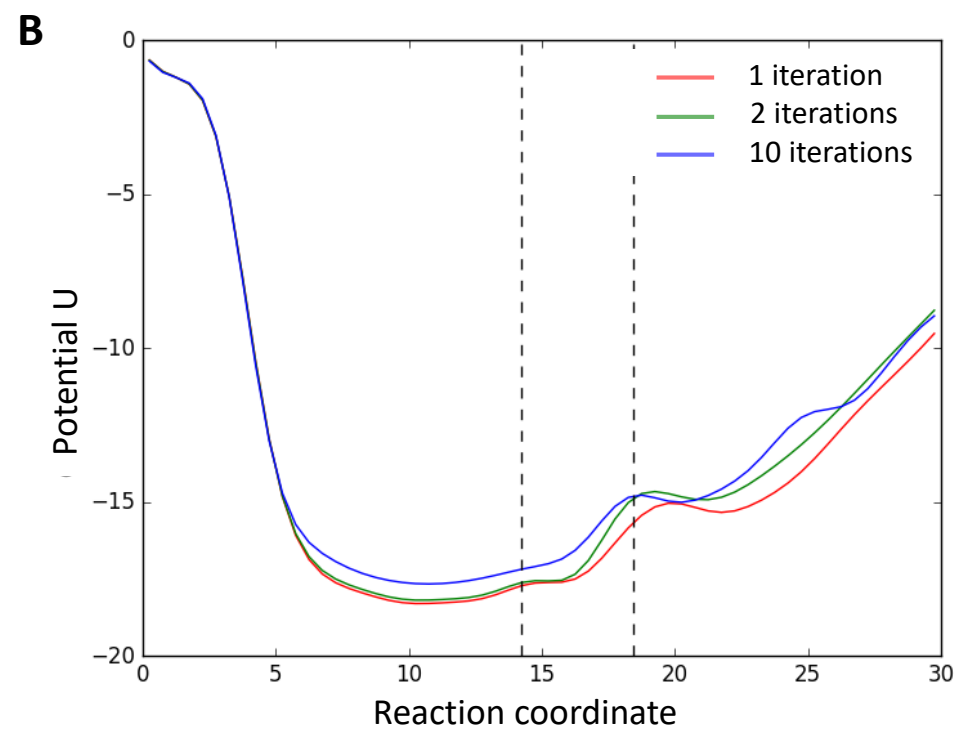

Supplement: S12 Fig — A. Three principal curves calculated with different iteration number. B. Potential U calculated for all three different principal curves. (PDF) [file pcbi.1007034.s012.pdf]
