# Supplementary material for: Phenotypic heterogeneity and evolution of melanoma cells associated with targeted therapy resistance
Source: PLoS Comput Biol. 2019 Jun 5;15(6):e1007034. doi: 10.1371/journal.pcbi.1007034 (PMC6576794; doi:10.1371/journal.pcbi.1007034)
Supplement: S6 Fig — (PDF) [file pcbi.1007034.s006.pdf]

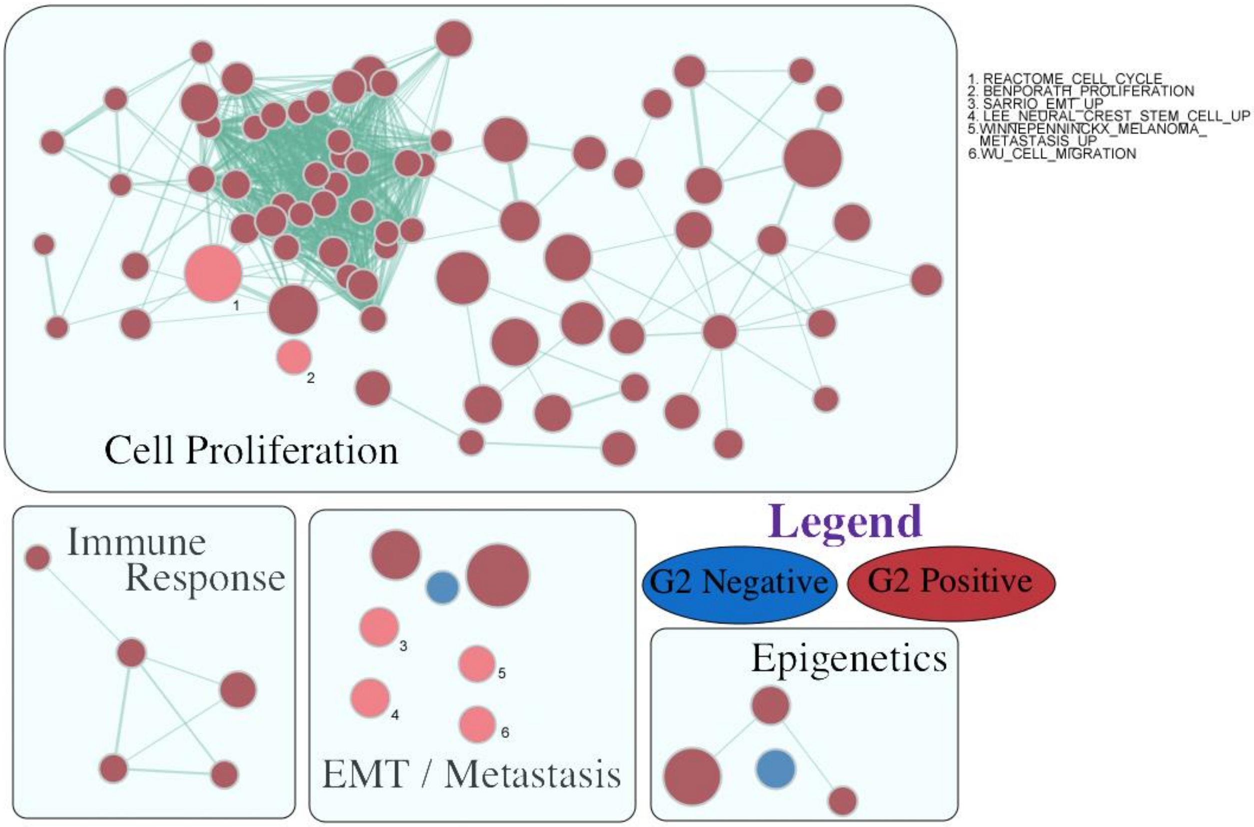

1. REACTOME_CELL_CYCLE
2. BENPORATH_PROLIFERATION
3. SARRIO_EMT_UP
4. LEE_NEURAL_CREST_STEM_CELL_UP
5.WINNEPENNINCKX_MELANOMA_
METASTASIS_UP
6.WU_CELL_MIGRATION
Cell Proliferation
Immune Response
EMT / Metastasis
Legend
G2 Negative
G2 Positive
Epigenetics
